# Forecast of peak attainment and imminent decline after 2017 of oral cancer incidence in men in Taiwan

**DOI:** 10.1038/s41598-022-09736-2

**Published:** 2022-04-06

**Authors:** Jing-Rong Jhuang, Shih-Yung Su, Chun-Ju Chiang, Ya-Wen Yang, Li-Ju Lin, Tsui-Hsia Hsu, Wen-Chung Lee

**Affiliations:** 1 Institute of Epidemiology and Preventive Medicine, College of Public Health, National Taiwan University, Rm. 536, No. 17, Xuzhou Rd., Taipei, 100 Taiwan; 2 Innovation and Policy Center for Population Health and Sustainable Environment, College of Public Health, National Taiwan University, Taipei, Taiwan; 3 Taiwan Cancer Registry, Taipei, Taiwan; 4 Health Promotion Administration, Taipei, Taiwan

**Keywords:** Diseases, Oncology, Mathematics and computing

## Abstract

Oral cancer is the fourth most common cancer among men in Taiwan. The age-standardized incidence rate of oral cancer among men in Taiwan has increased since 1980 and became six times greater in 2014. To enable effective public health planning for oral cancer, research on the projection of oral cancer burden is essential. We conducted an age-period-cohort analysis on the incidence of oral cancer among men in Taiwan from 1997 to 2017 and extrapolated the trend to 2025. We found that the period trends for young adults aged between 25 and 44 have already peaked before 2017; the younger, the earlier, and then the trends declined. The cohort trends have peaked roughly at the 1972 birth cohort and then declined for all ages. Despite the increasing trend in the age-standardized incidence rate for oral cancer among men in Taiwan from 1997 to 2017, we forecast a peak attained, an imminent decline after 2017, and a decrease of 8.4% in age-standardized incidence rate from 2017 to 2025. The findings of this study contribute to developing efficient and comprehensive strategies for oral cancer prevention and control.

## Introduction

Worldwide increases in the incidence of various cancers impose a significant burden on health and economics^1^. A total of 14.1 and 18.1 million new cancer cases were diagnosed in 2012 and 2018, respectively, an increase of approximately 28.4%^2,3^. Among all cancers, oral cancer (including lip, oral cavity, oropharynx, and hypopharynx cancers) is a major disease category because of its substantial impact on patients’ quality of life^4–8^. From 1990 to 2017, the global incidence of oral cancer exhibited an upward trend, and increases were primarily for people aged 15–49 years and with low-middle socioeconomic status^9^. Men are more than twice as likely as women to develop oral cancer. In Taiwan, male predominance is even more notable; oral cancer is the fourth most common cancer among men with an incidence of 7,400 cases (42.15 per 100,000 people), but only the fifteenth common cancer among women with an incidence of 770 cases (3.92 per 100,000 people) in 2018^10^.

In recent years, the escalating global cancer burden motivates cancer incidence projections for cancer prevention and control^11–27^. Two studies regarding the prediction of oral cancer incidence were successively conducted in the U.S.^26,27^. The first one made projections with the joinpoint regression^28^, and their forecasts supported the argument that strengthened vaccination efforts of human papillomavirus virus (HPV) to reduce the future burden. The second one used the age-period-cohort (APC) model^29^ instead, given the strong cohort effect of oral cancer incidence in the U.S. They forecast a continued shift in the incidence to an older population because of the low HPV vaccination rates among older individuals. In Taiwan, the age-standardized incidence rate of oral cancer among men has increased since 1980, and in 2014 the incidence rate was sixfold that of 1980^30^. Such a rapid increase may be associated with cigarette smoking and betel quid chewing in the population^30–36^. To enable effective public health planning for oral cancer, research projecting the oral cancer burden is essential. Therefore, we studied the incidence trend of oral cancer among men in Taiwan from 1997 to 2017 and extrapolated the trend to 2025 by the APC model. We discussed possible factors driving these trends and suggested improving oral cancer prevention and control.

## Materials and methods

### Ethics

This study protocol was approved by the National Taiwan University Research Ethics Committee (202101HM030) and the Data Release Review Board of the Health Promotion Administration, Ministry of Health and Welfare in Taiwan. All methods were performed following the relevant guidelines and regulations. In addition, the Research Ethics Committee waived the requirement for informed consent due to the lack of personal information and secondary data in the study.

### Data source, case definition, and study population

All information on newly diagnosed malignant neoplasms from hospitals with capacities of > 50 beds has been recorded in the Taiwan Cancer Registry dataset, a nationwide, population-based registry, since 1979. Every patient in the dataset comprises demographics (sex, date of birth, and residential area code) and diagnostic data (date of diagnosis, tumor site, histopathological information, and tumor grade). To strengthen the validity, completeness, and timeliness of the Taiwan Cancer Registry Dataset, multiple verification processes were conducted, containing logical and consistency assessments, duplicate checks, and trace-back of death certificate-only cases. The registry has been in its maturity stage since 2003 and with stable high quality (timeliness < 14 months, completeness > 98%, a morphological verified rate ≈ 93%, and a percentage of cases registered only in death certificate < 1%)^37–39^. Cases with a definitive diagnosis before 2002 were encoded according to the *Field Trial Edition of the International Classification of Disease for Oncology* (ICD-O-FT). The newest version, ICD-O-3, has been used as the standard code source since 2003^40^. Yearly cancer incidence data (ICO-O-FT: 140, 141, 143–146, 148, 149; ICD-O-3: C00–C06, C09, C10, C12–C14) for men from 1997 to 2017 were included in the study.

Patients were categorized into twelve 5-year age groups (25–29, 30–34, 35–39, 40–44, 45–49, …, 80–84). We excluded patients aged < 25 years or > 84 years because of the scarcity of cases. The period between 1997 and 2017 was divided into twenty 1-year groups. The corresponding periods and age group population were obtained from the online database maintained by the Department of Statistics of Taiwan’s Ministry of the Interior. The World Health Organization 2000 World Standard Population with the truncated age interval of 25–84 was used for age standardization (Table S6).

### Age‑period‑cohort model

The APC model was used to analyze the oral cancer incidence rate and project the trend to 2025, given the strong cohort effect in Taiwan^36^. However, because of the perfect linearity of the temporal variables (cohort + age = period), an infinite set of parameter estimates with equal goodness of fit exists, causing the non-identifiability problem. In addition, we used data provided in 5-year age groups and 1-year periods in the study, which might cause additional identifiability issues with the unequal intervals in their definition of cohort indices^41^. To circumvent the non-identifiability problem inherent in the APC model, the linear cohort effect was not estimated in the APC model, presented as follows:

$${\text{g}}\left( {\frac{{\upmu }}{m}} \right) = {\uptheta } + {\upalpha }_{1} a + {\upbeta }_{1} p + {\text{f}}_{{\text{A}}} \left( a \right) + {\text{f}}_{{\text{P}}} \left( p \right) + {\text{f}}_{{\text{C}}} \left( c \right)$$

where g(.) was the link function, ${\upmu }$ was the expected incidence cases, *m* was the person-years, *a, p, and c* were the age variable, the period variable, and the cohort variable, respectively, ${\uptheta }$ was the intercept, ${\upalpha }_{1}$ and ${\upbeta }_{1}$ were the linear age effect and the linear period effect, respectively, and ${\text{f}}_{{\text{A}}} \left( a \right)$, ${\text{f}}_{{\text{P}}} \left( p \right)$, and ${\text{f}}_{{\text{C}}} \left( c \right)$ were functions of the age variable, the period variable, and the cohort variable, respectively, denoting the nonlinear effects. Maximum likelihood estimation was used for estimating the parameters in the APC model.

### Ensemble learning and model selection

We applied an ensemble technique to obtain an APC model with the best predictive performance. In this technique, various APC models in the ensemble are trained on the given training dataset. Finally, the model that performs best on the validation set is chosen for future use. We considered a threefold validation, splitting the complete data (21 calendar years from 1997 to 2017) into a training set (14 calendar years from 1997 to 2010) and a validation set (7 calendar years from 2011 to 2017). A total of 52 types of APC models (with the formula presented in Table S4) were considered for training. The model types referred to in previous research^11–13,42–45^, included polynomial APC prediction models (Type 1 to Type 17)^42^, Tzeng and Lee’s APC prediction model (Type 18 to Type 22)^43,44^, and cubic splines APC prediction models (Type 23 to Type 52)^11–13,45^. For the cubic splines APC models, the knot locations were placed at:

$$\left\{ {\begin{array}{ll} {\text{k}}_{a,i} = \frac{{{\text{n}}_{a} }}{{{\text{k}}_{a} + 1}} \times i, &\quad for\, i = 1,2, \ldots ,{\text{k}}_{a} \\ {\text{k}}_{{{\text{p}},j}} = \frac{{{\text{n}}_{p} }}{{{\text{k}}_{p} + 1}} \times j, &\quad for\, j = 1,2, \ldots ,{\text{k}}_{p} \\ {\text{k}}_{c,k} = \frac{{{\text{n}}_{c} }}{{{\text{k}}_{c} + 1}} \times k,&\quad for \,k = 1,2, \ldots ,{\text{k}}_{c} \\ \end{array} } \right.$$

where ${\text{n}}_{a}$, ${\text{n}}_{p}$, and ${\text{n}}_{c}$ were the number of age groups, period groups, and cohort groups, respectively, and ${\text{k}}_{a}$, ${\text{k}}_{p}$, and ${\text{k}}_{c}$ were the number of knots for age, period, and cohort, respectively. We considered five types of link functions (log, power 2, power 3, power 4, and power 5) for the 52 model types. Also, with the assumption that historical trends will not continue indefinitely^11–13,42^, each APC model projection was applied to 21 levels (0%, 5%, 10%, 15%, …, or 100%) of year-on-year attenuation (${\text{P}}^{*} = \left( {{\text{O}}_{{{\text{mark}}}} - {\text{P}}} \right) \times {\text{A}} + {\text{P}})$, where ${\text{P}}^{*}$ was the attenuated predicted value, ${\text{P}}$ was the predicted value, ${\text{O}}_{{{\text{mark}}}}$ was the last observed value, and A was the attenuation level). A total of 5460 models (52 $\times$ 5 $\times$ 21) were constructed and formed the ensemble APC model.

The ensemble APC model was used to predict the incidence rate of the validation set (calendar years from 2011 to 2017). Model selection was based on the index of the symmetric mean absolute percentage error (${\text{SMAPE}} = \frac{1}{12 \times 7}\sum\nolimits_{{{\text{p}} = 2011}}^{2017} {\sum\nolimits_{{{\text{a}} = 1}}^{12} {\frac{{\left| {{\text{P}}_{{{\text{p}},{\text{a}}}} - {\text{O}}_{{{\text{p}},{\text{a}}}} } \right|}}{{\left( {{\text{P}}_{{{\text{p}},{\text{a}}}} + {\text{O}}_{{{\text{p}},{\text{a}}}} } \right)/2}}} }$, where ${\text{P}}_{{{\text{p}},{\text{a}}}}$ was the predicted value and ${\text{O}}_{{{\text{p}},{\text{a}}}}$ was the observed value) and the logarithmic score (${\text{LOGS}} = \sum\nolimits_{{{\text{p}} = 2011}}^{2017} {\sum\nolimits_{{{\text{a}} = 1}}^{12} {\log } } \left( {\Pr \left( {{\text{O}}_{{{\text{p}},{\text{a}}}} } \right)} \right)$, where $\Pr \left( . \right)$ was the probability density function and ${\text{O}}_{{{\text{p}},{\text{a}}}}$ was the observed value). We selected a final model with the lowest SMAPE or the maximal LOGS. Finally, we re-estimated the model parameters based on oral cancer incidence data from 1997 to 2017 (all available data) and made projections for 2025. We presented a diagram of the method for building APC prediction models in Fig. S1.

We also conducted sensitivity analyses and presented results in the supplementary materials. The sensitivity analyses included working the model selection with a fivefold validation (16 calendar years from 1997 to 2012 for training and the remaining five calendar years for validating), applying log-transformation on the SMAPE (${\text{SMAPE}} - {\text{T}} = \frac{1}{12 \times 7}\sum\nolimits_{{{\text{p}} = 2011}}^{2017} {\sum\nolimits_{{{\text{a}} = 1}}^{12} {\log } } \left( {\frac{{\left| {{\text{P}}_{{{\text{p}},{\text{a}}}} - {\text{O}}_{{{\text{p}},{\text{a}}}} } \right|}}{{\left( {{\text{P}}_{{{\text{p}},{\text{a}}}} + {\text{O}}_{{{\text{p}},{\text{a}}}} } \right)/2}}} \right)$), considering another attenuation strategy with ${\text{P}}_{2011}^{*} = \left( {{\text{O}}_{{{\text{mark}}}} - {\text{P}}_{2011} } \right) \times 0{\text{\% }} + {\text{P}}_{2011}$ and ${\text{P}}_{{\text{t}}}^{*} = \left( {{\text{O}}_{{{\text{mark}}}} - {\text{P}}_{{\text{t}}} } \right) \times {\text{A}} + {\text{P}}_{{\text{t}}}$, where ${\text{t}} \ge 2012$, and performing the same analysis for women. All statistical analyses were performed using SAS version 9.4 (SAS Institute Inc, Cary, NC, USA). The SAS code for analysis was presented in the supplementary materials.

## Results

**Figure 1:**
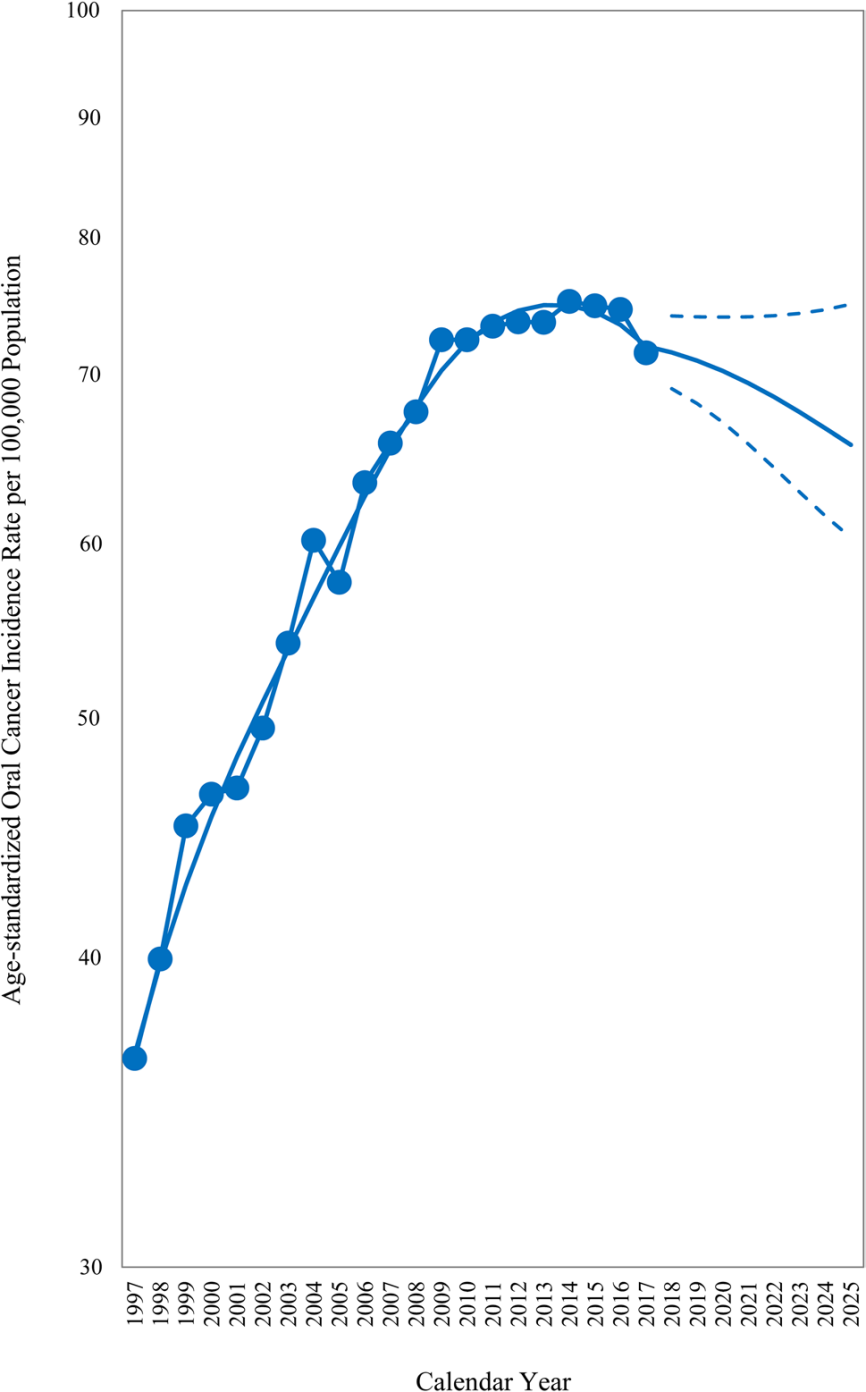
Age-standardized oral cancer incidence rates from 1997 to 2017 and the age–period–cohort model projections from 2018 to 2025. The World Health Organization 2000 Standard Population was used to compute the truncated age-standardized incidence rates (age range: 25–84 years), see Table S6.

We selected the optimal model as a cubic splines APC model (type 26 in Table S4) with 80% attenuation, presented as follows: ${\text{log}}\left( {\frac{{\upmu }}{m}} \right) = - 13.7585 + 2.5052a - 0.3793a^{2} + 0.0221a^{3} - 0.0146\left( {a - 4} \right)_{ + }^{3} - 0.0070\left( {a - 8} \right)_{ + }^{3} + 0.0364p - 0.0102p^{2} + 0.0005p^{3} - 0.0007\left( {p - 7} \right)_{ + }^{3} + 0.0006\left( {p - 14} \right)_{ + }^{3} + 0.0108c^{2} - 0.0005c^{3} + 0.0004\left( {c - 8} \right)_{ + }^{3} - 0.0001\left( {c - 16} \right)_{ + }^{3} - 0.0013\left( {c - 24} \right)_{ + }^{3}$ where $\left( x \right)_{ + } = {\text{max}}\left( {0,x} \right)$. The validation statistics of the selected model were SMAPE = 8.59 and LOGS = − 469.75. The age-standardized oral cancer incidence for 1997–2017 and projections by the APC model for 2018 to 2025 are presented in Fig. 1. The age-standardized incidence rate increased considerably from 1997, with the incidence rate doubled in 2009 compared with 1997, but then the increasing trend slowed. The projection indicated that the trend would start to decline in 2017 and further decline until 2025.

**Figure 2:**
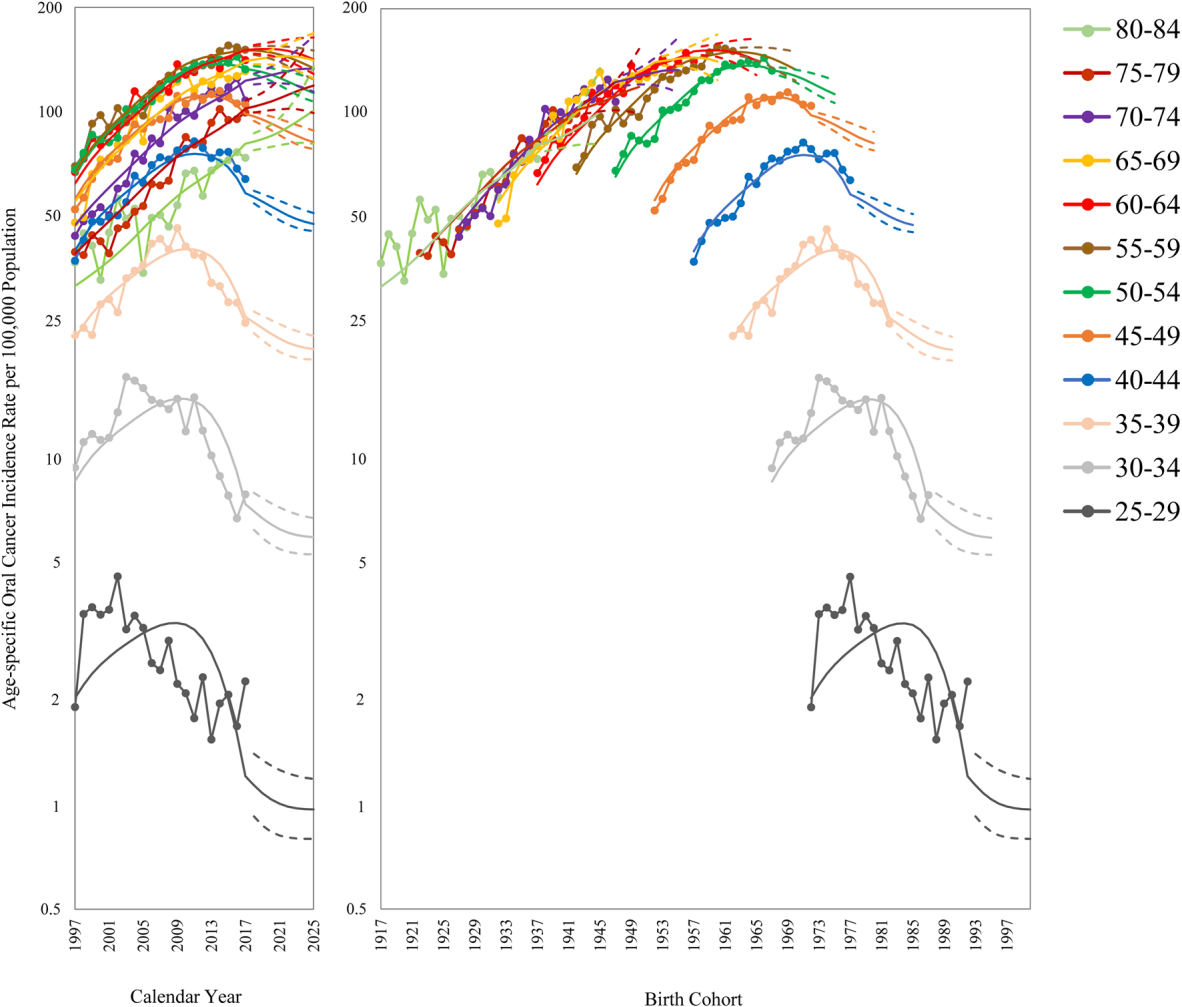
Age-specific oral cancer incidence rates among men in Taiwan by calendar year and birth cohort (age range: 25–84 years).

Data on age-specific oral cancer incidence rate among men in Taiwan by calendar year and birth cohort are displayed in Fig. 2. Period trends diverged across age groups. Period trends for older age groups (70–74, 75–79, and 80–84) were rapidly increasing, and the projections also show an increasing trend. Period trends for the middle-age groups (40–44, 45–49, 50–54, 55–59, 60–64, and 65–69) were increasing at first but then leveled off, with projections indicating a stable trend (60–64 and 65–69) or decrease (40–44, 45–49, 50–54, and 55–59). Period trends for the younger age groups (25–29, 30–34, and 35–39) initially increased rapidly, peaked—the younger, the earlier, and then decreased. Period trends for these younger age groups are projected to further decline until 2025. Birth cohort trends were consistent for all age groups: trends increased before 1973 and decreased after that.

The age-standardized and age-specific oral cancer incidence per 100,000 population (observed rates in 2017 and projected rates in 2025) and projected percentage changes from 2018 to 2025 are shown in Table 1. The age-standardized incidence rate is poised to decrease by 8.4% from 2017 to 2025. The age-specific incidence rates of age groups 80–84, 75–79, and 70–74 are predicted to increase substantially by $\ge$ 20% from 2017 to 2025. The age-specific incidence rates for 65–69 and 60–64 are expected to increase slightly by < 10% from 2017 to 2025. The age-specific incidence rate of 55–59 will decrease by approximately 10% from 2017 to 2025. The age-specific incidence rates of 50–54 and 35–39 will reduce by about 15% from 2017 to 2025. The age-specific incidence rates of the remaining age groups (45–49, 40–44, 30–34, and 25–29) are expected to decrease substantially by > 20% from 2017 to 2025.

**Table 1:** Age-standardized and age-specific oral cancer incidence per 100,000 population (observed rates in 2017 and projected rates for 2025) and projected percentage changes from 2018 to 2025.

	Incidence in 2017 per 100,000 population	Projected incidence in 2025 per 100,000 population [95% Uncertainty Interval]	Projected percentage change from 2018 [95% Uncertainty Interval]
Age-standardized rates^#^	72.05	65.98 [60.36, 75.49]	− 8.42 [− 16.21, 4.78]
**Age-specific rates**			
Age 25–29	2.27	0.97 [0.80, 1.19]	− 57.32 [− 64.89, − 47.71]
Age 30–34	7.85	5.92 [5.28, 6.72]	− 24.66 [− 32.79, − 14.51]
Age 35–39	24.58	20.63 [19.24, 22.53]	− 16.05 [− 21.73, − 8.34]
Age 40–44	63.86	47.39 [45.17, 50.93]	− 25.78 [− 29.26, − 20.25]
Age 45–49	104.74	81.60 [77.83, 88.18]	− 22.09 [− 25.69, − 15.81]
Age 50–54	132.40	113.10 [106.74, 124.25]	− 14.57 [− 19.38, − 6.15]
Age 55–59	150.77	133.43 [123.84, 150.01]	− 11.50 [− 17.86, − 0.50]
Age 60–64	140.67	141.42 [128.47, 163.60]	0.53 [− 8.68, 16.30]
Age 65–69	130.79	140.66 [124.42, 168.38]	7.55 [− 4.87, 28.74]
Age 70–74	107.56	132.79 [113.90, 165.02]	23.45 [5.89, 53.41]
Age 75–79	99.43	118.81 [99.05, 152.85]	19.49 [− 0.39, 53.73]
Age 80–84	73.54	100.59 [80.77, 134.50]	36.79 [9.83, 82.90]

^#^The World Health Organization 2000 World Standard Population was used to compute the truncated age-standardized incidence rates (age 25–84 years), see Table S6.

The results of the sensitivity analysis were presented in the supplementary materials. The optimal model based on 0% attenuation in 2011 and 80% attenuation from 2012 to 2017 was the type 26 model with ${\text{SMAPE}} = 9.04$ and ${\text{LOGS}} = - 486.84$. The results for men based on the SMAPE-T (Fig. S3) or a 5-fold validation (Figs.S6-S7) showed similar projected trends. In addition, the projection for women (Figs. S4-S5) showed a similar age-standardized incidence trend to men. However, the age-specific incidence rate among women increased slower than among men for 65–84.

## Discussion

The age-standardized incidence rate of oral cancer among men in Taiwan presented a rapidly increasing trend at first, but the increasing trend slowed after 2009. The historical trend of age-standardized rate alone makes it challenging to visualize peak attainment and imminent decline after 2017. By contrast, the APC analysis showed that period trends for young adults aged 25–44 years had already peaked before 2017—the younger, the earlier, and then the trends declined. Moreover, a significant cohort effect was revealed, decreasing incidence rates at roughly the 1972 birth cohort for all ages. The combined results indicate a peak has been attained, and the trend is now for oral cancer incidence to decrease among men in Taiwan. Previously, Hsu et al.^30^ predicted a future decline but not before 2025. However, they only created one APC model and did not use techniques to prevent overfitting. By contrast, we used a data-splitting method and constructed an ensemble APC model to allow for flexible model specification. Furthermore, Hsu et al.^30^ only studied the trend in age-standardized incidence rate but not the period trends and birth cohort trends by age.

We did not estimate the linear cohort effect for making parameters identifiable in the study. The use of additional constraints can successfully decompose the APC effect. However, different constraints may cause drastically different or even contradictory results. There is no consensus in the APC literature on which constraints are the best and used. Besides, the incidence projections in this study were impervious to the non-identifiability problem because the fitted values in the nonidentifiable APC model were the same for all possible sets of parameter estimates. According to Figs. 1 and 2, a discontinuity could be observed when the prediction started, caused by imposing the 80% attenuation on the predictive value. The idea of attenuating the projected trend comes from the belief that historical trends will not continue indefinitely and was shown to be valuable empirically for making future predictions by Møller et al.^11–13,42^ When alleviating the attenuation level from 80 to 0%, the result showed a rapidly decreasing trend after 2017 (Fig. S8). Still, the SMAPE would increase from 8.59 to 11.52, and the LOGS would drop from − 469.75 to − 490.82. With the experience by Møller et al.^13,42^, we also found that it worked better to gradually reduce the projected trend (SMAPE = 8.59 and LOGS = − 469.75) than attenuate the trend a period after the last observation (SMAPE = 9.04 and LOGS = − 486.84). Therefore, it seems favorable to gradually attenuate the projected trend in the study. In addition, the selected type 26 model with 80% attenuation had the lowest SMAPE (8.59) but the second-highest LOGS (− 469.75). Notably, the same model with a bit higher 85% attenuation had the second-lowest SMAPE (8.78) and the highest LOGS (− 460.61). Therefore, it is suitable to predict the oral cancer incidence among men in Taiwan with the type 26 model with 80% or 85% attenuation.

The data splitting method in the study was referred to in the previous studies^20,25^ in Taiwan. However, unlike a 2-fold validation in those studies, we applied a 3-fold validation, two-thirds of the calendar years (1997–2010) used for training and the remaining (2011–2017) for validating, for the following motivation and reasons: (1) given the calendar year ended in 2010, we could include enough data cells for the 1972 birth cohort and later for training (Table S3) to capture the cohort effect for oral cancer among men in Taiwan^36^, (2) a sensitivity analysis with a 5-fold validation was considered, and the similar projected trends were obtained (Figs. S6-S7), and (3) Taiwan have implemented the National Health Insurance Program and passed the legislation of Tobacco Hazards Prevention Act since 1997, which might impact the oral cancer incidence trend in Taiwan.

Betel quid chewing and smoking are risk factors for oral cancer among men in Taiwan^46,47^. In 2005 in Taiwan, the betel quid chewing rate was approximately 10%^48^, and the smoking rate among men was about 50%^49^. Notably, the betel quid chewing rate for the indigenous peoples of Taiwan was four times the overall rate in Taiwan^50^. The World Health Organization Global Oral Health Programme presented the common risk factor approach for preventing and controlling non-communicable diseases^51,52^. Public health interventions, including tax policies and health education^49,53–56^, for preventing excessive risk exposure of these lifestyle factors^57^ have been implemented since 1997 in Taiwan. These measures have continuously declined Taiwan's betel quid chewing and smoking rates^49,54^. Su et al.^36^ conducted an APC analysis to examine the incidence rate of oral cancer among men in Taiwan from 1997 to 2016. They found a strong association between the cohort effect on oral cancer incidence rate among men and average betel nut consumption with a lag time of 30 years. A decrease in smoking rates may also contribute to the decreasing oral cancer incidence rate after the 1972 birth cohort. Per our projections, by 2025, the age-specific incidence rate will have decreased for those between 25 and 59 years old but increased for those between 60 and 84 years old. Health care professionals have actively implemented long-term care services since 2007 to alleviate the excessive burden of an aging population in Taiwan to ensure older adults can live healthier lives^58,59^.

Early detection of oral cancer can prolong life expectancy^60^. Taiwan started a national oral cancer screening program in 2004^61,62^ that provides a free oral mucosal examination every two years for Taiwanese residents with habits of smoking or betel quid chewing. Between 2004 and 2009, the overall oral cancer screening rate was 55.1% in Taiwan^62^. A shift has been observed, indicating that patients with malignant oral cancer can be detected earlier because of the screening program^62^. In addition, we obtained contrasting projected oral incidence trends in men and women for 65–84. Because men might have a higher prevalence of risk factors and more opportunities to be screened for oral cancer, the early detection due to the screening program might have caused the increase in oral cancer incidence among men aged between 65 and 84 since 2004.

In conclusion, despite the increasing trend in the age-standardized incidence rate of oral cancer among men in Taiwan from 1997 to 2017, we determined that a peak was reached in 2017, and the incidence has subsequently been in decline, with a decrease of 8.4% in age-standardized incidence from 2017 to 2025. Therefore, the findings of this study contribute to developing efficient and comprehensive strategies for oral cancer prevention and control.

## Supplementary Information

Supplementary Information.

## Data Availability

The authors confirm that all data underlying the findings are fully available without restriction. The data underlying the results of this study are either available in the manuscript or upon request from the corresponding author, Wen-Chung Lee. The raw data cannot be made available in supplemental files or a public repository because of privacy or ethical reasons. However, the dataset used for analysis can be obtained in an anonymized form upon request from the corresponding author.

## Abbreviations

APC: Age-period-cohort
HPV: Human papillomavirus virus
SMAPE: Symmetric mean absolute percentage error
