# Supplementary material for: Forecast of peak attainment and imminent decline after 2017 of oral cancer incidence in men in Taiwan
Source: Sci Rep. 2022 Apr 6;12:5726. doi: 10.1038/s41598-022-09736-2 (PMC8987068; doi:10.1038/s41598-022-09736-2)
Supplement: Supplementary file 1 — Supplementary Information. [file 41598_2022_9736_MOESM1_ESM.pdf]

Forecast of Peak Attainment and Imminent Decline after 2017 of Oral Cancer Incidence in Men in Taiwan

Authorship: Jing-Rong Jhuang[1,3], Shih-Yung Su[1,2], Chun-Ju Chiang[1,3], Ya-Wen Yang[1,3], Li-Ju Lin[4], Tsui-Hsia Hsu[4], Wen-Chung Lee[1,2,3*]

Affiliations:

[1] Institute of Epidemiology and Preventive Medicine, College of Public Health, National Taiwan University, Taipei, Taiwan

[2] Innovation and Policy Center for Population Health and Sustainable Environment, College of Public Health, National Taiwan University, Taipei, Taiwan

[3] Taiwan Cancer Registry, Taipei, Taiwan

[4] Health Promotion Administration, Taipei, Taiwan

[*]**Corresponding authors:** Prof. Wen-Chung Lee,

Rm. 536, No. 17, Xuzhou Rd.,

Taipei 100, Taiwan.

(FAX: 886-2-23511955)

(e-mail: wenchung@ntu.edu.tw)

Abbreviations: ASPIR, Age-specific Incidence Rate; ASTIR, Age-standardized Incidence Rate; LOGS, Logarithmic Score; SMAPE, Symmetric Mean Absolute Percentage Error; SMAPE-T, Symmetric Mean Absolute Percentage Error-Transformed; UI, Uncertainty Interval.

Table S1. Observed ASTIR per 100,000 population, predicted ASTIR per 100,000 population, and 95% UI.

Calendar Year	Observed ASTIR#	Predicted ASTIR#	95% UI
1997	36.64	36.80	-
1998	40.30	40.15	-
1999	45.79	43.29	-
2000	47.20	46.20	-
2001	47.47	48.94	-
2002	50.29	51.56	-
2003	54.55	54.21	-
2004	60.20	56.99	-
2005	57.82	59.88	-
2006	63.62	62.81	-
2007	66.07	65.70	-
2008	68.08	68.42	-
2009	72.95	70.84	-
2010	72.95	72.80	-
2011	73.91	74.18	-
2012	74.21	75.02	-
2013	74.18	75.41	-
2014	75.68	75.39	-
2015	75.36	74.93	-
2016	75.10	74.00	-
2017	72.05	72.50	-
2018	-	72.07	[69.61-74.64]
2019	-	71.50	[68.62-74.57]
2020	-	70.81	[67.40-74.55]
2021	-	69.99	[66.01-74.58]
2022	-	69.06	[64.54-74.66]
2023	-	68.07	[63.05-74.83]
2024	-	67.03	[61.64-75.09]
2025	-	65.98	[60.36-75.49]

#The World Health Organization 2000 World Standard Population was used to compute the truncated age-standardized incidence rates (age 25–84 years), see Table S6.

Table S2. Observed ASPIR per 100,000 population, predicted ASPIR per 100,000 population, and 95% UI.

Calendar Year	25-29 years			30-34 years			35-39 years		
	Observed ASPIR	Predicted ASPIR	95% UI	Observed ASPIR	Predicted ASPIR	95% UI	Observed ASPIR	Predicted ASPIR	95% UI
1997	1.91	2.03	-	9.38	8.60	-	22.66	22.16	-
1998	3.55	2.22	-	11.12	9.40	-	23.76	24.26	-
1999	3.71	2.39	-	11.74	10.13	-	22.66	26.17	-
2000	3.53	2.54	-	11.28	10.77	-	27.77	27.88	-
2001	3.66	2.67	-	11.45	11.36	-	28.73	29.44	-
2002	4.56	2.79	-	13.56	11.90	-	26.35	30.89	-
2003	3.21	2.91	-	17.14	12.43	-	33.02	32.33	-
2004	3.51	3.02	-	16.74	12.96	-	34.69	33.81	-
2005	3.24	3.13	-	15.92	13.48	-	36.06	35.30	-
2006	2.56	3.22	-	14.72	13.96	-	41.59	36.74	-
2007	2.45	3.29	-	14.40	14.37	-	42.95	38.03	-
2008	2.97	3.34	-	13.83	14.67	-	39.97	39.09	-
2009	2.23	3.34	-	14.84	14.84	-	46.04	39.83	-
2010	2.10	3.31	-	11.96	14.83	-	40.81	40.16	-
2011	1.78	3.20	-	14.98	14.62	-	38.60	40.01	-
2012	2.33	3.01	-	12.01	14.14	-	38.13	39.40	-
2013	1.54	2.75	-	10.17	13.34	-	32.02	38.18	-
2014	1.96	2.41	-	8.88	12.21	-	31.35	36.20	-
2015	2.08	2.01	-	7.80	10.78	-	28.18	33.38	-
2016	1.69	1.61	-	6.70	9.12	-	28.08	29.78	-
2017	2.27	1.21	-	7.85	7.37	-	24.58	25.56	-
2018	-	1.14	0.93-1.40	-	7.02	6.21-7.94	-	24.64	22.93-26.49
2019	-	1.08	0.87-1.35	-	6.71	5.89-7.67	-	23.72	21.89-25.75
2020	-	1.04	0.84-1.30	-	6.44	5.64-7.42	-	22.86	20.98-25.04
2021	-	1.01	0.82-1.26	-	6.24	5.47-7.21	-	22.12	20.27-24.37
2022	-	0.99	0.80-1.23	-	6.10	5.37-7.03	-	21.53	19.78-23.78
2023	-	0.98	0.80-1.21	-	6.00	5.31-6.89	-	21.10	19.48-23.27
2024	-	0.97	0.80-1.20	-	5.95	5.29-6.79	-	20.81	19.31-22.86
2025	-	0.97	0.80-1.19	-	5.92	5.28-6.72	-	20.63	19.24-22.53

Table S2 (Continued). Observed ASPIR per 100,000 population, predicted ASPIR per 100,000 population, and 95% UI.

Calendar Year	40-44 years			45-49 years			50-54 years		
	Observed ASPIR	Predicted ASPIR	95% UI	Observed ASPIR	Predicted ASPIR	95% UI	Observed ASPIR	Predicted ASPIR	95% UI
1997	37.09	39.76	-	52.19	55.90	-	68.00	65.36	-
1998	42.55	43.59	-	56.41	61.33	-	76.00	71.72	-
1999	48.06	47.06	-	63.94	66.28	-	85.75	77.58	-
2000	48.09	50.20	-	69.93	70.79	-	83.13	82.94	-
2001	49.62	53.08	-	71.58	74.94	-	81.45	87.91	-
2002	50.10	55.80	-	72.99	78.89	-	83.95	92.66	-
2003	54.75	58.50	-	83.49	82.83	-	101.50	97.43	-
2004	65.32	61.30	-	91.76	86.95	-	101.85	102.44	-
2005	62.19	64.18	-	89.25	91.23	-	103.48	107.66	-
2006	70.04	67.04	-	93.38	95.56	-	106.91	112.99	-
2007	73.74	69.73	-	95.04	99.77	-	115.37	118.30	-
2008	72.74	72.09	-	95.71	103.63	-	124.11	123.35	-
2009	77.24	73.95	-	110.93	106.91	-	124.19	127.86	-
2010	78.10	75.12	-	105.20	109.35	-	131.38	131.52	-
2011	82.01	75.50	-	110.49	110.74	-	137.75	134.09	-
2012	78.53	75.16	-	108.00	111.20	-	136.72	135.68	-
2013	73.46	74.16	-	112.18	110.92	-	138.97	136.51	-
2014	76.22	72.21	-	114.63	109.97	-	141.17	136.82	-
2015	76.44	68.99	-	110.50	107.90	-	138.32	136.70	-
2016	68.42	64.30	-	105.50	104.17	-	144.04	135.55	-
2017	63.86	58.14	-	104.74	98.41	-	132.40	132.64	-
2018	-	56.65	54.35-59.07	-	96.81	93.95-99.77	-	131.58	128.01-135.26
2019	-	55.00	52.36-57.83	-	94.81	91.39-98.41	-	129.97	125.70-134.44
2020	-	53.28	50.37-56.55	-	92.48	88.47-96.87	-	127.79	122.66-133.35
2021	-	51.63	48.60-55.27	-	89.97	85.49-95.20	-	125.12	119.10-131.99
2022	-	50.16	47.18-54.02	-	87.48	82.77-93.43	-	122.10	115.39-130.36
2023	-	48.95	46.17-52.86	-	85.16	80.53-91.63	-	118.93	111.90-128.49
2024	-	48.03	45.53-51.82	-	83.18	78.90-89.86	-	115.86	108.96-126.43
2025	-	47.39	45.17-50.93	-	81.60	77.83-88.18	-	113.10	106.74-124.25

Table S2 (Continued). Observed ASPIR per 100,000 population, predicted ASPIR per 100,000 population, and 95% UI.

Calendar Year	55-59 years			60-64 years			65-69 years		
	Observed ASPIR	Predicted ASPIR	95% UI	Observed ASPIR	Predicted ASPIR	95% UI	Observed ASPIR	Predicted ASPIR	95% UI
1997	69.52	66.62	-	66.92	62.06	-	47.81	55.03	-
1998	75.03	72.95	-	72.99	67.63	-	49.46	59.51	-
1999	92.25	78.93	-	83.67	73.02	-	66.03	63.94	-
2000	97.64	84.46	-	79.95	78.16	-	72.59	68.30	-
2001	89.92	89.61	-	87.79	83.00	-	73.25	72.55	-
2002	102.26	94.56	-	90.21	87.67	-	80.09	76.70	-
2003	93.06	99.55	-	96.60	92.41	-	82.37	80.92	-
2004	100.61	104.81	-	114.34	97.42	-	98.15	85.41	-
2005	97.33	110.33	-	107.80	102.69	-	81.94	90.15	-
2006	109.92	115.99	-	113.31	108.13	-	107.77	95.06	-
2007	116.57	121.69	-	119.91	113.63	-	108.83	100.05	-
2008	126.97	127.24	-	113.86	119.06	-	114.97	105.01	-
2009	124.86	132.39	-	136.97	124.23	-	121.82	109.79	-
2010	130.16	136.83	-	127.61	128.88	-	131.13	114.22	-
2011	132.55	140.31	-	129.90	132.78	-	114.62	118.13	-
2012	135.80	142.92	-	136.44	136.03	-	122.18	121.59	-
2013	136.00	144.90	-	142.75	138.84	-	119.68	124.81	-
2014	149.53	146.49	-	133.25	141.44	-	127.91	128.00	-
2015	155.22	147.95	-	140.56	144.09	-	124.25	131.41	-
2016	153.30	149.39	-	148.94	147.08	-	126.48	135.29	-
2017	150.77	150.13	-	140.67	150.51	-	130.79	139.96	-
2018	-	149.97	145.85-154.22	-	151.16	146.88-155.58	-	141.08	136.78-145.53
2019	-	149.33	144.37-154.53	-	151.58	146.34-157.08	-	142.28	137.00-147.84
2020	-	148.10	142.05-154.62	-	151.60	145.09-158.64	-	143.39	136.73-150.60
2021	-	146.18	138.91-154.42	-	151.03	143.00-160.14	-	144.19	135.80-153.75
2022	-	143.61	135.16-153.89	-	149.74	140.07-161.48	-	144.49	134.07-157.20
2023	-	140.48	131.12-152.98	-	147.66	136.45-162.57	-	144.09	131.50-160.86
2024	-	137.01	127.22-151.69	-	144.84	132.45-163.30	-	142.83	128.20-164.63
2025	-	133.43	123.84-150.01	-	141.42	128.47-163.60	-	140.66	124.42-168.38

Table S2 (Continued). Observed ASPIR per 100,000 population, predicted ASPIR per 100,000 population, and 95% UI.

Calendar Year	70-74 years			75-79 years			80-84 years		
	Observed ASPIR	Predicted ASPIR	95% UI	Observed ASPIR	Predicted ASPIR	95% UI	Observed ASPIR	Predicted ASPIR	95% UI
1997	43.82	47.02	-	39.31	38.93	-	36.68	31.39	-
1998	48.25	50.32	-	38.54	41.11	-	44.59	32.62	-
1999	50.52	53.66	-	44.00	43.38	-	41.04	33.97	-
2000	52.96	57.03	-	42.24	45.76	-	32.66	35.46	-
2001	50.26	60.46	-	38.97	48.27	-	44.76	37.12	-
2002	59.92	63.93	-	46.01	50.94	-	56.07	38.98	-
2003	61.97	67.51	-	47.05	53.80	-	49.03	41.09	-
2004	75.55	71.32	-	51.41	56.89	-	52.45	43.46	-
2005	72.14	75.37	-	53.34	60.18	-	34.20	46.01	-
2006	83.61	79.58	-	61.80	63.61	-	49.38	48.68	-
2007	80.99	83.88	-	61.23	67.13	-	50.29	51.44	-
2008	102.51	88.17	-	63.16	70.67	-	46.58	54.22	-
2009	96.10	92.34	-	76.27	74.13	-	53.63	56.95	-
2010	100.41	96.26	-	84.39	77.41	-	66.31	59.57	-
2011	97.19	99.83	-	80.28	80.44	-	67.49	62.01	-
2012	109.76	103.15	-	81.54	83.35	-	57.13	64.38	-
2013	116.99	106.39	-	92.80	86.29	-	67.55	66.84	-
2014	109.23	109.74	-	101.63	89.43	-	71.35	69.53	-
2015	117.54	113.41	-	94.49	92.95	-	72.37	72.61	-
2016	124.70	117.66	-	95.17	97.09	-	76.33	76.28	-
2017	107.56	122.77	-	99.43	102.08	-	73.54	80.74	-
2018	-	124.04	119.67-128.57	-	103.32	99.37-107.43	-	81.85	77.39-86.57
2019	-	125.59	120.35-131.12	-	104.88	100.17-109.84	-	83.25	78.23-88.61
2020	-	127.36	120.78-134.50	-	106.82	100.94-113.22	-	85.03	79.09-91.54
2021	-	129.17	120.80-138.73	-	109.11	101.55-117.76	-	87.31	79.94-95.71
2022	-	130.86	120.22-143.86	-	111.65	101.83-123.69	-	90.07	80.69-101.50
2023	-	132.19	118.90-149.93	-	114.26	101.59-131.28	-	93.29	81.19-109.41
2024	-	132.91	116.78-156.98	-	116.74	100.69-140.87	-	96.85	81.26-120.11
2025	-	132.79	113.90-165.02	-	118.81	99.05-152.85	-	100.59	80.77-134.50

Table S3. Data cells used for the training set and the validation set.

Period \ Age	25-29	30-34	35-39	40-44	45-49	50-54	55-59	60-64	65-69	70-74	75-79	80-84
**Training set**												
1997	1972	1967	1962	1957	1952	1947	1942	1937	1932	1927	1922	1917
1998	1973	1968	1963	1958	1953	1948	1943	1938	1933	1928	1923	1918
1999	1974	1969	1964	1959	1954	1949	1944	1939	1934	1929	1924	1919
2000	1975	1970	1965	1960	1955	1950	1945	1940	1935	1930	1925	1920
2001	1976	1971	1966	1961	1956	1951	1946	1941	1936	1931	1926	1921
2002	1977	1972	1967	1962	1957	1952	1947	1942	1937	1932	1927	1922
2003	1978	1973	1968	1963	1958	1953	1948	1943	1938	1933	1928	1923
2004	1979	1974	1969	1964	1959	1954	1949	1944	1939	1934	1929	1924
2005	1980	1975	1970	1965	1960	1955	1950	1945	1940	1935	1930	1925
2006	1981	1976	1971	1966	1961	1956	1951	1946	1941	1936	1931	1926
2007	1982	1977	1972	1967	1962	1957	1952	1947	1942	1937	1932	1927
2008	1983	1978	1973	1968	1963	1958	1953	1948	1943	1938	1933	1928
2009	1984	1979	1974	1969	1964	1959	1954	1949	1944	1939	1934	1929
2010	1985	1980	1975	1970	1965	1960	1955	1950	1945	1940	1935	1930
**Validation set**												
2011	1986	1981	1976	1971	1966	1961	1956	1951	1946	1941	1936	1931
2012	1987	1982	1977	1972	1967	1962	1957	1952	1947	1942	1937	1932
2013	1988	1983	1978	1973	1968	1963	1958	1953	1948	1943	1938	1933
2014	1989	1984	1979	1974	1969	1964	1959	1954	1949	1944	1939	1934
2015	1990	1985	1980	1975	1970	1965	1960	1955	1950	1945	1940	1935
2016	1991	1986	1981	1976	1971	1966	1961	1956	1951	1946	1941	1936
2017	1992	1987	1982	1977	1972	1967	1962	1957	1952	1947	1942	1937

Note: For the 1972 birth cohort and later, around half of the data cells were used for the training set. (27/55=49%)

Table S4. 52 model types in the ensemble.

Type	Polynomial APC prediction models	Type	Tzeng and Lee's APC prediction model
Type 1	$g\left(\frac{\mu}{m}\right) = \theta + \alpha_1 a + \alpha_2 a^2 + \beta_1 p + \beta_2 p^2 + \gamma_2 c^2$	Type 18	$g\left(\frac{\mu}{m}\right) = \theta + \alpha_L a_L + \boldsymbol{\alpha}_{\mathbf{C}}^{\mathbf{T}} \boldsymbol{a}_{\boldsymbol{C}} + \beta_1 p + \beta_2 p^2 + \beta_3 p^3 + \gamma_2 c^2 + \gamma_3 c^3$
Type 2	$g\left(\frac{\mu}{m}\right) = \theta + \alpha_1 a + \alpha_2 a^2 + \beta_1 p + \gamma_2 c^2$	Type 19	$g\left(\frac{\mu}{m}\right) = \theta + \alpha_L a_L + \boldsymbol{\alpha}_{\mathbf{C}}^{\mathbf{T}} \boldsymbol{a}_{\boldsymbol{C}} + \beta_1 p + \beta_2 p^2 + \beta_3 p^3 + \gamma_2 c^2$
Type 3	$g\left(\frac{\mu}{m}\right) = \theta + \alpha_1 a + \alpha_2 a^2 + \alpha_3 a^3 + \beta_1 p + \beta_2 p^2 + \beta_3 p^3 + \gamma_2 c^2 + \gamma_3 c^3$	Type 20	$g\left(\frac{\mu}{m}\right) = \theta + \alpha_L a_L + \boldsymbol{\alpha}_{\mathbf{C}}^{\mathbf{T}} \boldsymbol{a}_{\boldsymbol{C}} + \beta_1 p + \beta_2 p^2 + \gamma_2 c^2 + \gamma_3 c^3$
Type 4	$g\left(\frac{\mu}{m}\right) = \theta + \alpha_1 a + \alpha_2 a^2 + \alpha_3 a^3 + \beta_1 p + \beta_2 p^2 + \beta_3 p^3 + \gamma_2 c^2$	Type 21	$g\left(\frac{\mu}{m}\right) = \theta + \alpha_L a_L + \boldsymbol{\alpha}_{\mathbf{C}}^{\mathbf{T}} \boldsymbol{a}_{\boldsymbol{C}} + \beta_1 p + \beta_2 p^2 + \gamma_2 c^2$
Type 5	$g\left(\frac{\mu}{m}\right) = \theta + \alpha_1 a + \alpha_2 a^2 + \alpha_3 a^3 + \beta_1 p + \beta_2 p^2 + \gamma_2 c^2 + \gamma_3 c^3$	Type 22	$g\left(\frac{\mu}{m}\right) = \theta + \alpha_L a_L + \boldsymbol{\alpha}_{\mathbf{C}}^{\mathbf{T}} \boldsymbol{a}_{\boldsymbol{C}} + \beta_1 p + \gamma_2 c^2$
Type 6	$g\left(\frac{\mu}{m}\right) = \theta + \alpha_1 a + \alpha_2 a^2 + \alpha_3 a^3 + \beta_1 p + \beta_2 p^2 + \gamma_2 c^2$		
Type 7	$g\left(\frac{\mu}{m}\right) = \theta + \alpha_1 a + \alpha_2 a^2 + \alpha_3 a^3 + \beta_1 p + \gamma_2 c^2$		
Type 8	$g\left(\frac{\mu}{m}\right) = \theta + \alpha_1 a + \alpha_2 a^2 + \alpha_3 a^3 + \alpha_4 a^4 + \beta_1 p + \beta_2 p^2 + \beta_3 p^3 + \gamma_2 c^2 + \gamma_3 c^3$		
Type 9	$g\left(\frac{\mu}{m}\right) = \theta + \alpha_1 a + \alpha_2 a^2 + \alpha_3 a^3 + \alpha_4 a^4 + \beta_1 p + \beta_2 p^2 + \beta_3 p^3 + \gamma_2 c^2$		
Type 10	$g\left(\frac{\mu}{m}\right) = \theta + \alpha_1 a + \alpha_2 a^2 + \alpha_3 a^3 + \alpha_4 a^4 + \beta_1 p + \beta_2 p^2 + \gamma_2 c^2 + \gamma_3 c^3$		
Type 11	$g\left(\frac{\mu}{m}\right) = \theta + \alpha_1 a + \alpha_2 a^2 + \alpha_3 a^3 + \alpha_4 a^4 + \beta_1 p + \beta_2 p^2 + \gamma_2 c^2$		
Type 12	$g\left(\frac{\mu}{m}\right) = \theta + \alpha_1 a + \alpha_2 a^2 + \alpha_3 a^3 + \alpha_4 a^4 + \beta_1 p + \gamma_2 c^2$		
Type 13	$g\left(\frac{\mu}{m}\right) = \theta + \alpha_1 a + \alpha_2 a^2 + \alpha_3 a^3 + \alpha_4 a^4 + \alpha_5 a^5 + \beta_1 p + \beta_2 p^2 + \beta_3 p^3 + \gamma_2 c^2 + \gamma_3 c^3$		
Type 14	$g\left(\frac{\mu}{m}\right) = \theta + \alpha_1 a + \alpha_2 a^2 + \alpha_3 a^3 + \alpha_4 a^4 + \alpha_5 a^5 + \beta_1 p + \beta_2 p^2 + \beta_3 p^3 + \gamma_2 c^2$		
Type 15	$g\left(\frac{\mu}{m}\right) = \theta + \alpha_1 a + \alpha_2 a^2 + \alpha_3 a^3 + \alpha_4 a^4 + \alpha_5 a^5 + \beta_1 p + \beta_2 p^2 + \gamma_2 c^2 + \gamma_3 c^3$		
Type 16	$g\left(\frac{\mu}{m}\right) = \theta + \alpha_1 a + \alpha_2 a^2 + \alpha_3 a^3 + \alpha_4 a^4 + \alpha_5 a^5 + \beta_1 p + \beta_2 p^2 + \gamma_2 c^2$		
Type 17	$g\left(\frac{\mu}{m}\right) = \theta + \alpha_1 a + \alpha_2 a^2 + \alpha_3 a^3 + \alpha_4 a^4 + \alpha_5 a^5 + \beta_1 p + \gamma_2 c^2$		

Model notation: g(.), the link function; μ, the expected incidence cases; *m*, the person-years; *a*, the age variable; *p*, the period variable; *c*, the cohort variable; $(a_L, \boldsymbol{a}_{\boldsymbol{C}})$, the linear and curvature components partitioned from the age variable; $(p_L, \boldsymbol{p}_{\boldsymbol{C}})$, the linear and curvature components partitioned from the period variable; $\boldsymbol{c}_{\boldsymbol{C}}$, the curvature component partitioned from the cohort variable; θ, the intercept; $\alpha_1 - \alpha_5$, the polynomial effects of age; $\beta_1 - \beta_3$, the polynomial effects of period; $\gamma_2 - \gamma_3$, the polynomial effects of cohort; $(\alpha_L, \boldsymbol{\alpha}_{\mathbf{C}})$, the linear trend and curvature effects of age; $(\beta_L, \boldsymbol{\beta}_{\mathbf{C}})$, the linear trend and curvature effects of period; $\boldsymbol{\gamma}_{\mathbf{C}}$, the curvature effects of cohort.

Table S4 (Continued). 52 model types in the ensemble.

Type	Cubic splines APC prediction model	Type	Cubic splines APC prediction model
Type 23	$\text{Type 3} + \sum_{i=1}^{2} \alpha_{3+i}(a - k_{a,i})_+^3 + \sum_{i=1}^{2} \beta_{3+i}(p - k_{p,i})_+^3 + \sum_{i=1}^{2} \gamma_{3+i}(c - k_{c,i})_+^3$	Type 39	$\text{Type 3} + \sum_{i=1}^{2} \alpha_{3+i}(a - k_{a,i})_+^3 + \sum_{i=1}^{3} \beta_{3+i}(p - k_{p,i})_+^3 + \sum_{i=1}^{2} \gamma_{3+i}(c - k_{c,i})_+^3$
Type 24	$\text{Type 3} + \sum_{i=1}^{3} \alpha_{3+i}(a - k_{a,i})_+^3 + \sum_{i=1}^{2} \beta_{3+i}(p - k_{p,i})_+^3 + \sum_{i=1}^{2} \gamma_{3+i}(c - k_{c,i})_+^3$	Type 39	$\text{Type 3} + \sum_{i=1}^{3} \alpha_{3+i}(a - k_{a,i})_+^3 + \sum_{i=1}^{3} \beta_{3+i}(p - k_{p,i})_+^3 + \sum_{i=1}^{2} \gamma_{3+i}(c - k_{c,i})_+^3$
Type 25	$\text{Type 3} + \sum_{i=1}^{4} \alpha_{3+i}(a - k_{a,i})_+^3 + \sum_{i=1}^{2} \beta_{3+i}(p - k_{p,i})_+^3 + \sum_{i=1}^{2} \gamma_{3+i}(c - k_{c,i})_+^3$	Type 40	$\text{Type 3} + \sum_{i=1}^{4} \alpha_{3+i}(a - k_{a,i})_+^3 + \sum_{i=1}^{3} \beta_{3+i}(p - k_{p,i})_+^3 + \sum_{i=1}^{2} \gamma_{3+i}(c - k_{c,i})_+^3$
Type 26	$\text{Type 3} + \sum_{i=1}^{2} \alpha_{3+i}(a - k_{a,i})_+^3 + \sum_{i=1}^{2} \beta_{3+i}(p - k_{p,i})_+^3 + \sum_{i=1}^{3} \gamma_{3+i}(c - k_{c,i})_+^3$	Type 41	$\text{Type 3} + \sum_{i=1}^{2} \alpha_{3+i}(a - k_{a,i})_+^3 + \sum_{i=1}^{3} \beta_{3+i}(p - k_{p,i})_+^3 + \sum_{i=1}^{3} \gamma_{3+i}(c - k_{c,i})_+^3$
Type 27	$\text{Type 3} + \sum_{i=1}^{3} \alpha_{3+i}(a - k_{a,i})_+^3 + \sum_{i=1}^{2} \beta_{3+i}(p - k_{p,i})_+^3 + \sum_{i=1}^{3} \gamma_{3+i}(c - k_{c,i})_+^3$	Type 42	$\text{Type 3} + \sum_{i=1}^{3} \alpha_{3+i}(a - k_{a,i})_+^3 + \sum_{i=1}^{3} \beta_{3+i}(p - k_{p,i})_+^3 + \sum_{i=1}^{3} \gamma_{3+i}(c - k_{c,i})_+^3$
Type 28	$\text{Type 3} + \sum_{i=1}^{4} \alpha_{3+i}(a - k_{a,i})_+^3 + \sum_{i=1}^{2} \beta_{3+i}(p - k_{p,i})_+^3 + \sum_{i=1}^{3} \gamma_{3+i}(c - k_{c,i})_+^3$	Type 43	$\text{Type 3} + \sum_{i=1}^{4} \alpha_{3+i}(a - k_{a,i})_+^3 + \sum_{i=1}^{3} \beta_{3+i}(p - k_{p,i})_+^3 + \sum_{i=1}^{3} \gamma_{3+i}(c - k_{c,i})_+^3$
Type 29	$\text{Type 3} + \sum_{i=1}^{2} \alpha_{3+i}(a - k_{a,i})_+^3 + \sum_{i=1}^{2} \beta_{3+i}(p - k_{p,i})_+^3 + \sum_{i=1}^{4} \gamma_{3+i}(c - k_{c,i})_+^3$	Type 44	$\text{Type 3} + \sum_{i=1}^{2} \alpha_{3+i}(a - k_{a,i})_+^3 + \sum_{i=1}^{3} \beta_{3+i}(p - k_{p,i})_+^3 + \sum_{i=1}^{4} \gamma_{3+i}(c - k_{c,i})_+^3$
Type 30	$\text{Type 3} + \sum_{i=1}^{3} \alpha_{3+i}(a - k_{a,i})_+^3 + \sum_{i=1}^{2} \beta_{3+i}(p - k_{p,i})_+^3 + \sum_{i=1}^{4} \gamma_{3+i}(c - k_{c,i})_+^3$	Type 45	$\text{Type 3} + \sum_{i=1}^{3} \alpha_{3+i}(a - k_{a,i})_+^3 + \sum_{i=1}^{3} \beta_{3+i}(p - k_{p,i})_+^3 + \sum_{i=1}^{4} \gamma_{3+i}(c - k_{c,i})_+^3$
Type 31	$\text{Type 3} + \sum_{i=1}^{4} \alpha_{3+i}(a - k_{a,i})_+^3 + \sum_{i=1}^{2} \beta_{3+i}(p - k_{p,i})_+^3 + \sum_{i=1}^{4} \gamma_{3+i}(c - k_{c,i})_+^3$	Type 46	$\text{Type 3} + \sum_{i=1}^{4} \alpha_{3+i}(a - k_{a,i})_+^3 + \sum_{i=1}^{3} \beta_{3+i}(p - k_{p,i})_+^3 + \sum_{i=1}^{4} \gamma_{3+i}(c - k_{c,i})_+^3$
Type 32	$\text{Type 3} + \sum_{i=1}^{2} \alpha_{3+i}(a - k_{a,i})_+^3 + \sum_{i=1}^{2} \beta_{3+i}(p - k_{p,i})_+^3 + \sum_{i=1}^{5} \gamma_{3+i}(c - k_{c,i})_+^3$	Type 47	$\text{Type 3} + \sum_{i=1}^{2} \alpha_{3+i}(a - k_{a,i})_+^3 + \sum_{i=1}^{3} \beta_{3+i}(p - k_{p,i})_+^3 + \sum_{i=1}^{5} \gamma_{3+i}(c - k_{c,i})_+^3$
Type 33	$\text{Type 3} + \sum_{i=1}^{3} \alpha_{3+i}(a - k_{a,i})_+^3 + \sum_{i=1}^{2} \beta_{3+i}(p - k_{p,i})_+^3 + \sum_{i=1}^{5} \gamma_{3+i}(c - k_{c,i})_+^3$	Type 48	$\text{Type 3} + \sum_{i=1}^{3} \alpha_{3+i}(a - k_{a,i})_+^3 + \sum_{i=1}^{3} \beta_{3+i}(p - k_{p,i})_+^3 + \sum_{i=1}^{5} \gamma_{3+i}(c - k_{c,i})_+^3$
Type 34	$\text{Type 3} + \sum_{i=1}^{4} \alpha_{3+i}(a - k_{a,i})_+^3 + \sum_{i=1}^{2} \beta_{3+i}(p - k_{p,i})_+^3 + \sum_{i=1}^{5} \gamma_{3+i}(c - k_{c,i})_+^3$	Type 49	$\text{Type 3} + \sum_{i=1}^{4} \alpha_{3+i}(a - k_{a,i})_+^3 + \sum_{i=1}^{3} \beta_{3+i}(p - k_{p,i})_+^3 + \sum_{i=1}^{5} \gamma_{3+i}(c - k_{c,i})_+^3$
Type 35	$\text{Type 3} + \sum_{i=1}^{2} \alpha_{3+i}(a - k_{a,i})_+^3 + \sum_{i=1}^{2} \beta_{3+i}(p - k_{p,i})_+^3 + \sum_{i=1}^{6} \gamma_{3+i}(c - k_{c,i})_+^3$	Type 50	$\text{Type 3} + \sum_{i=1}^{2} \alpha_{3+i}(a - k_{a,i})_+^3 + \sum_{i=1}^{3} \beta_{3+i}(p - k_{p,i})_+^3 + \sum_{i=1}^{6} \gamma_{3+i}(c - k_{c,i})_+^3$
Type 36	$\text{Type 3} + \sum_{i=1}^{3} \alpha_{3+i}(a - k_{a,i})_+^3 + \sum_{i=1}^{2} \beta_{3+i}(p - k_{p,i})_+^3 + \sum_{i=1}^{6} \gamma_{3+i}(c - k_{c,i})_+^3$	Type 51	$\text{Type 3} + \sum_{i=1}^{3} \alpha_{3+i}(a - k_{a,i})_+^3 + \sum_{i=1}^{3} \beta_{3+i}(p - k_{p,i})_+^3 + \sum_{i=1}^{6} \gamma_{3+i}(c - k_{c,i})_+^3$
Type 37	$\text{Type 3} + \sum_{i=1}^{4} \alpha_{3+i}(a - k_{a,i})_+^3 + \sum_{i=1}^{2} \beta_{3+i}(p - k_{p,i})_+^3 + \sum_{i=1}^{6} \gamma_{3+i}(c - k_{c,i})_+^3$	Type 52	$\text{Type 3} + \sum_{i=1}^{4} \alpha_{3+i}(a - k_{a,i})_+^3 + \sum_{i=1}^{3} \beta_{3+i}(p - k_{p,i})_+^3 + \sum_{i=1}^{6} \gamma_{3+i}(c - k_{c,i})_+^3$

Model notation: Type 3, the type 3 model in Table S4; *a*, the age variable; *p*, the period variable; *c*, the cohort variable; $(x)_+ = \max(0, x)$; $\alpha_4 - \alpha_7$, the cubic splines effects of age; $\beta_4 - \beta_6$, the cubic splines effects of period; $\gamma_4 - \gamma_9$, the cubic splines effects of cohort. The knot locations were placed at:

$$\begin{cases} k_{a,i} = \frac{n_a}{k_a+1} \times i, \text{ for } i = 1,2,\ldots,k_a \\ k_{p,j} = \frac{n_p}{k_p+1} \times j, \text{ for } j = 1,2,\ldots,k_p \\ k_{c,k} = \frac{n_c}{k_c+1} \times k, \text{ for } k = 1,2,\ldots,k_c \end{cases}$$

where n_a, n_p, and n_c were the number of age groups, period groups, and period groups, respectively, and k_a, k_p, and k_c were the number of knots for age, period, and cohort, respectively.

Table S5. Validation statistics for the 52 model types.

Link	SPMAPE (Attenuation)	LOGS (Attenuation)	SPMAPE (Attenuation)	LOGS (Attenuation)	SPMAPE (Attenuation)	LOGS (Attenuation)
	**Type 1**		**Type 5**		**Type 9**	
log	29.56 (35%)	-1192.33 (65%)	13.31 (0%)	-508.76 (0%)	18.10 (65%)	-740.19 (80%)
power 2	24.21 (60%)	359.46 (65%)	16.91 (70%)	401.23 (75%)	23.95 (85%)	340.82 (90%)
power 3	22.28 (40%)	292.71 (55%)	12.75 (85%)	340.84 (75%)	20.57 (80%)	264.52 (90%)
power 4	21.56 (30%)	262.50 (45%)	11.66 (80%)	317.47 (75%)	19.01 (80%)	231.30 (90%)
power 5	21.55 (20%)	247.03 (30%)	11.31 (75%)	306.43 (80%)	18.11 (75%)	215.09 (95%)
	**Type 2**		**Type 6**		**Type 10**	
log	29.48 (60%)	-1185.74 (80%)	18.74 (45%)	-746.03 (65%)	11.52 (0%)	-490.82 (0%)
power 2	23.22 (0%)	368.82 (0%)	22.91 (65%)	352.12 (65%)	12.01 (95%)	411.90 (80%)
power 3	21.56 (0%)	301.08 (0%)	19.58 (45%)	279.54 (55%)	11.09 (75%)	348.44 (80%)
power 4	21.10 (0%)	269.08 (0%)	17.82 (30%)	248.74 (45%)	10.89 (75%)	321.17 (80%)
power 5	21.25 (0%)	252.09 (0%)	16.72 (20%)	235.16 (30%)	10.92 (75%)	308.11 (80%)
	**Type 3**		**Type 7**		**Type 11**	
log	15.63 (75%)	-599.57 (70%)	18.52 (65%)	-734.30 (80%)	17.82 (45%)	-729.21 (65%)
power 2	17.79 (95%)	404.92 (90%)	21.70 (0%)	362.69 (0%)	23.21 (55%)	349.41 (65%)
power 3	11.18 (90%)	346.83 (90%)	18.83 (0%)	292.61 (0%)	19.54 (40%)	276.99 (55%)
power 4	10.16 (90%)	323.22 (90%)	17.34 (0%)	261.91 (0%)	17.77 (30%)	247.52 (45%)
power 5	10.00 (90%)	311.53 (90%)	16.44 (0%)	247.01 (0%)	16.69 (10%)	234.77 (30%)
	**Type 4**		**Type 8**		**Type 12**	
log	19.03 (65%)	-758.19 (80%)	14.39 (65%)	-584.75 (70%)	17.66 (60%)	-719.57 (80%)
power 2	23.76 (80%)	346.34 (90%)	10.99 (90%)	426.87 (90%)	22.28 (0%)	365.36 (0%)
power 3	20.63 (75%)	269.20 (90%)	10.31 (90%)	358.14 (90%)	18.88 (0%)	292.76 (0%)
power 4	19.00 (75%)	233.78 (90%)	10.15 (90%)	328.41 (90%)	17.31 (0%)	261.79 (0%)
power 5	18.09 (75%)	216.11 (95%)	10.08 (90%)	313.74 (90%)	16.42 (0%)	247.01 (0%)

Table S5 (continued). Validation statistics for the 52 model types.

Link	SPMAPE (Attenuation)	LOGS (Attenuation)	SPMAPE (Attenuation)	LOGS (Attenuation)	SPMAPE (Attenuation)	LOGS (Attenuation)
	**Type 13**		**Type 17**		**Type 21**	
log	14.26 (65%)	-613.86 (70%)	17.33 (65%)	-746.64 (80%)	17.88 (50%)	-764.59 (70%)
power 2	11.07 (90%)	425.88 (90%)	22.31 (0%)	364.71 (0%)	23.35 (60%)	346.54 (65%)
power 3	10.60 (90%)	353.19 (90%)	19.07 (0%)	289.35 (0%)	19.92 (45%)	271.70 (55%)
power 4	10.66 (90%)	320.18 (90%)	17.67 (0%)	255.85 (0%)	18.35 (30%)	240.00 (45%)
power 5	10.73 (90%)	303.22 (90%)	16.92 (0%)	239.14 (0%)	17.42 (15%)	225.54 (30%)
	**Type 14**		**Type 18**		**Type 22**	
log	17.88 (65%)	-770.29 (80%)	14.71 (65%)	-618.17 (70%)	17.66 (65%)	-752.60 (80%)
power 2	23.98 (85%)	340.15 (90%)	11.03 (90%)	424.16 (90%)	22.38 (0%)	362.89 (0%)
power 3	20.71 (80%)	260.94 (90%)	10.71 (90%)	352.32 (90%)	19.17 (0%)	287.92 (0%)
power 4	19.34 (75%)	224.94 (90%)	10.82 (90%)	319.71 (90%)	17.78 (0%)	254.74 (0%)
power 5	18.58 (75%)	206.53 (95%)	10.90 (90%)	302.94 (90%)	17.05 (0%)	238.25 (0%)
	**Type 15**		**Type 19**		**Type 23**	
log	11.77 (0%)	-522.62 (0%)	18.12 (65%)	-774.52 (80%)	8.83 (75%)	-459.98 (80%)
power 2	12.08 (95%)	411.03 (80%)	24.05 (90%)	337.77 (90%)	11.24 (90%)	430.55 (90%)
power 3	11.43 (80%)	343.83 (80%)	20.88 (80%)	258.91 (90%)	9.97 (95%)	361.08 (95%)
power 4	11.42 (75%)	313.46 (80%)	19.48 (80%)	223.27 (90%)	9.65 (95%)	331.22 (95%)
power 5	11.54 (75%)	298.25 (80%)	18.72 (80%)	205.12 (95%)	9.57 (95%)	316.22 (95%)
	**Type 16**		**Type 20**		**Type 24**	
log	17.56 (45%)	-759.48 (65%)	12.19 (0%)	-529.93 (0%)	9.27 (75%)	-471.14 (80%)
power 2	23.23 (55%)	348.74 (65%)	11.88 (95%)	408.79 (80%)	11.35 (90%)	426.94 (90%)
power 3	19.71 (45%)	273.48 (55%)	11.21 (75%)	342.72 (80%)	10.16 (95%)	357.78 (95%)
power 4	18.14 (30%)	241.37 (45%)	11.29 (75%)	312.85 (80%)	9.92 (95%)	328.40 (95%)
power 5	17.24 (20%)	226.63 (30%)	11.44 (75%)	297.89 (80%)	9.84 (95%)	313.74 (95%)

Table S5 (continued). Validation statistics for the 52 model types.

Link	SPMAPE (Attenuation)	LOGS (Attenuation)	SPMAPE (Attenuation)	LOGS (Attenuation)	SPMAPE (Attenuation)	LOGS (Attenuation)
	**Type 25**		**Type 29**		**Type 33**	
log	9.37 (75%)	-470.66 (80%)	9.12 (85%)	-490.95 (90%)	10.54 (90%)	-531.46 (90%)
power 2	11.33 (90%)	427.91 (90%)	10.91 (95%)	425.23 (95%)	11.10 (95%)	422.33 (90%)
power 3	10.13 (95%)	358.38 (95%)	10.04 (95%)	359.95 (95%)	10.26 (95%)	358.28 (90%)
power 4	9.84 (95%)	328.83 (95%)	9.72 (95%)	330.75 (95%)	9.97 (95%)	328.23 (95%)
power 5	9.75 (95%)	314.08 (95%)	9.62 (95%)	315.95 (95%)	9.91 (95%)	313.55 (95%)
	**Type 26**		**Type 30**		**Type 34**	
log	8.59 (80%)	-460.61 (85%)	9.33 (85%)	-499.64 (90%)	10.70 (90%)	-532.60 (90%)
power 2	11.19 (95%)	426.98 (90%)	11.03 (95%)	421.41 (95%)	11.06 (95%)	423.92 (90%)
power 3	10.03 (95%)	360.14 (95%)	10.24 (95%)	356.40 (95%)	10.22 (95%)	358.95 (90%)
power 4	9.71 (95%)	330.60 (95%)	9.98 (95%)	327.74 (95%)	9.89 (95%)	328.70 (95%)
power 5	9.61 (95%)	315.77 (95%)	9.90 (95%)	313.31 (95%)	9.80 (95%)	313.90 (95%)
	**Type 27**		**Type 31**		**Type 35**	
log	8.93 (80%)	-470.95 (85%)	9.49 (85%)	-499.95 (90%)	10.14 (85%)	-512.93 (90%)
power 2	11.30 (95%)	423.23 (90%)	10.98 (95%)	422.43 (95%)	10.80 (95%)	422.42 (95%)
power 3	10.23 (95%)	356.64 (95%)	10.21 (95%)	357.06 (95%)	10.04 (95%)	359.75 (95%)
power 4	9.98 (95%)	327.57 (95%)	9.90 (95%)	328.21 (95%)	9.67 (95%)	331.29 (95%)
power 5	9.90 (95%)	313.08 (95%)	9.81 (95%)	313.67 (95%)	9.56 (95%)	316.68 (95%)
	**Type 28**		**Type 32**		**Type 36**	
log	9.08 (80%)	-471.03 (85%)	10.56 (85%)	-521.30 (90%)	10.12 (85%)	-521.69 (90%)
power 2	11.26 (95%)	424.16 (90%)	10.98 (95%)	426.42 (90%)	11.06 (95%)	417.98 (95%)
power 3	10.21 (95%)	357.24 (95%)	10.04 (95%)	361.55 (90%)	10.25 (95%)	355.89 (95%)
power 4	9.90 (95%)	327.99 (95%)	9.67 (95%)	331.45 (95%)	9.95 (95%)	328.01 (95%)
power 5	9.81 (95%)	313.40 (95%)	9.60 (95%)	316.41 (95%)	9.87 (95%)	313.78 (95%)

Table S5 (continued). Validation statistics for the 52 model types.

Link	SPMAPE (Attenuation)	LOGS (Attenuation)	SPMAPE (Attenuation)	LOGS (Attenuation)	SPMAPE (Attenuation)	LOGS (Attenuation)
	**Type 37**		**Type 41**		**Type 45**	
log	10.37 (90%)	-523.95 (90%)	11.36 (85%)	-558.73 (90%)	11.50 (90%)	-570.28 (95%)
power 2	10.92 (95%)	419.51 (95%)	11.50 (95%)	425.93 (85%)	10.83 (90%)	422.99 (80%)
power 3	10.22 (95%)	356.68 (95%)	10.55 (95%)	363.19 (75%)	10.56 (90%)	358.53 (85%)
power 4	9.90 (95%)	328.48 (95%)	9.81 (85%)	338.02 (85%)	9.99 (90%)	334.52 (85%)
power 5	9.77 (95%)	314.11 (95%)	9.71 (90%)	324.30 (85%)	9.90 (90%)	321.69 (85%)
	**Type 38**		**Type 42**		**Type 46**	
log	11.52 (70%)	-568.45 (90%)	11.39 (85%)	-565.37 (95%)	11.45 (90%)	-570.58 (95%)
power 2	12.25 (95%)	421.78 (90%)	11.59 (95%)	422.29 (85%)	10.79 (90%)	424.13 (80%)
power 3	10.72 (100%)	365.82 (85%)	10.63 (95%)	359.80 (75%)	10.50 (95%)	359.02 (85%)
power 4	9.77 (90%)	338.34 (85%)	9.98 (85%)	335.05 (85%)	9.89 (85%)	335.06 (85%)
power 5	9.69 (90%)	324.15 (85%)	9.90 (90%)	321.70 (85%)	9.77 (90%)	322.11 (85%)
	**Type 39**		**Type 43**		**Type 47**	
log	11.52 (70%)	-573.79 (90%)	11.36 (85%)	-565.69 (90%)	12.18 (95%)	-582.60 (95%)
power 2	12.32 (95%)	418.09 (90%)	11.47 (95%)	423.28 (85%)	10.82 (85%)	426.30 (80%)
power 3	10.76 (100%)	362.57 (85%)	10.55 (95%)	360.41 (75%)	10.81 (100%)	362.63 (85%)
power 4	9.89 (90%)	335.61 (85%)	9.87 (85%)	335.48 (85%)	9.60 (85%)	336.74 (85%)
power 5	9.85 (90%)	321.79 (85%)	9.77 (90%)	322.04 (85%)	9.90 (90%)	321.22 (85%)
	**Type 40**		**Type 44**		**Type 48**	
log	11.49 (70%)	-572.86 (90%)	11.46 (95%)	-562.91 (95%)	12.06 (95%)	-591.19 (95%)
power 2	12.22 (95%)	419.02 (90%)	10.85 (90%)	427.11 (80%)	10.75 (85%)	421.95 (80%)
power 3	10.70 (100%)	363.18 (85%)	10.51 (95%)	361.25 (85%)	10.86 (100%)	358.05 (80%)
power 4	9.79 (90%)	336.06 (85%)	9.83 (85%)	337.74 (85%)	9.81 (85%)	333.39 (85%)
power 5	9.73 (90%)	322.15 (85%)	9.70 (90%)	324.45 (85%)	10.11 (90%)	318.32 (85%)

Table S5 (continued). Validation statistics for the 52 model types.

Link	SPMAPE (Attenuation)	LOGS (Attenuation)
	**Type 49**	
log	12.08 (95%)	-591.43 (95%)
power 2	10.79 (90%)	423.22 (80%)
power 3	10.79 (100%)	359.39 (85%)
power 4	9.72 (85%)	333.88 (85%)
power 5	9.99 (90%)	318.58 (85%)
	**Type 50**	
log	11.86 (95%)	-574.80 (95%)
power 2	11.12 (90%)	424.79 (80%)
power 3	10.21 (90%)	366.13 (80%)
power 4	9.72 (85%)	339.42 (80%)
power 5	9.62 (90%)	325.47 (85%)
	**Type 51**	
log	11.74 (95%)	-582.23 (95%)
power 2	11.13 (90%)	420.19 (80%)
power 3	10.30 (90%)	362.72 (80%)
power 4	9.93 (85%)	336.35 (80%)
power 5	9.87 (90%)	322.77 (85%)
	**Type 52**	
log	11.77 (95%)	-583.50 (95%)
power 2	11.11 (90%)	422.08 (80%)
power 3	10.25 (90%)	363.02 (80%)
power 4	9.81 (85%)	336.86 (80%)
power 5	9.74 (90%)	323.07 (85%)

Table S6. The World Health Organization 2000 World Standard Population with the truncated age interval of 25–84.

Age interval	Standard population	Truncated population
0-4	8,800	0
5-9	8,700	0
10-14	8,600	0
15-19	8,500	0
20-24	8,200	0
25-29	7,900	13,958
30-34	7,600	13,428
35-39	7,200	12,721
40-44	6,600	11,661
45-49	6,000	10,600
50-54	5,400	9,541
55-59	4,600	8,127
60-64	3,700	6,537
65-69	3,000	5,300
70-74	2,200	3,887
75-79	1,500	2,650
80-84	900	1,590
85+	600	0
Total	100,000	100,000

Figure S1. Diagram of the method for building APC prediction models.

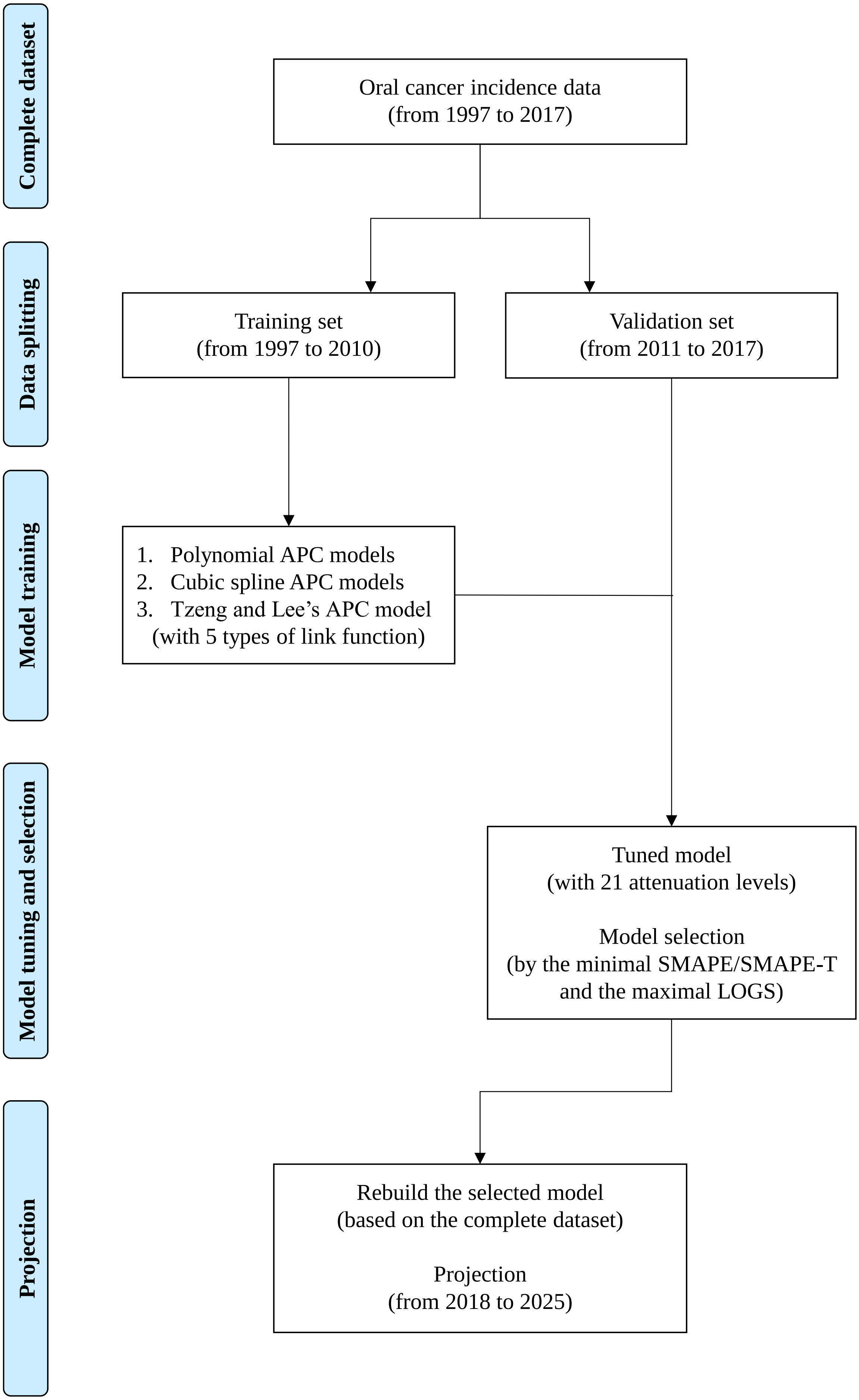

Figure S2. Age-specific oral cancer incidence rates among men in Taiwan by calendar year and birth cohort (age range: 40–84 years). The optimal model (from the 3-fold validation, SMAPE = 8.59, and LOGS = -469.75) was a restricted cubic spline model with a log link, type 26, and 80% attenuation).

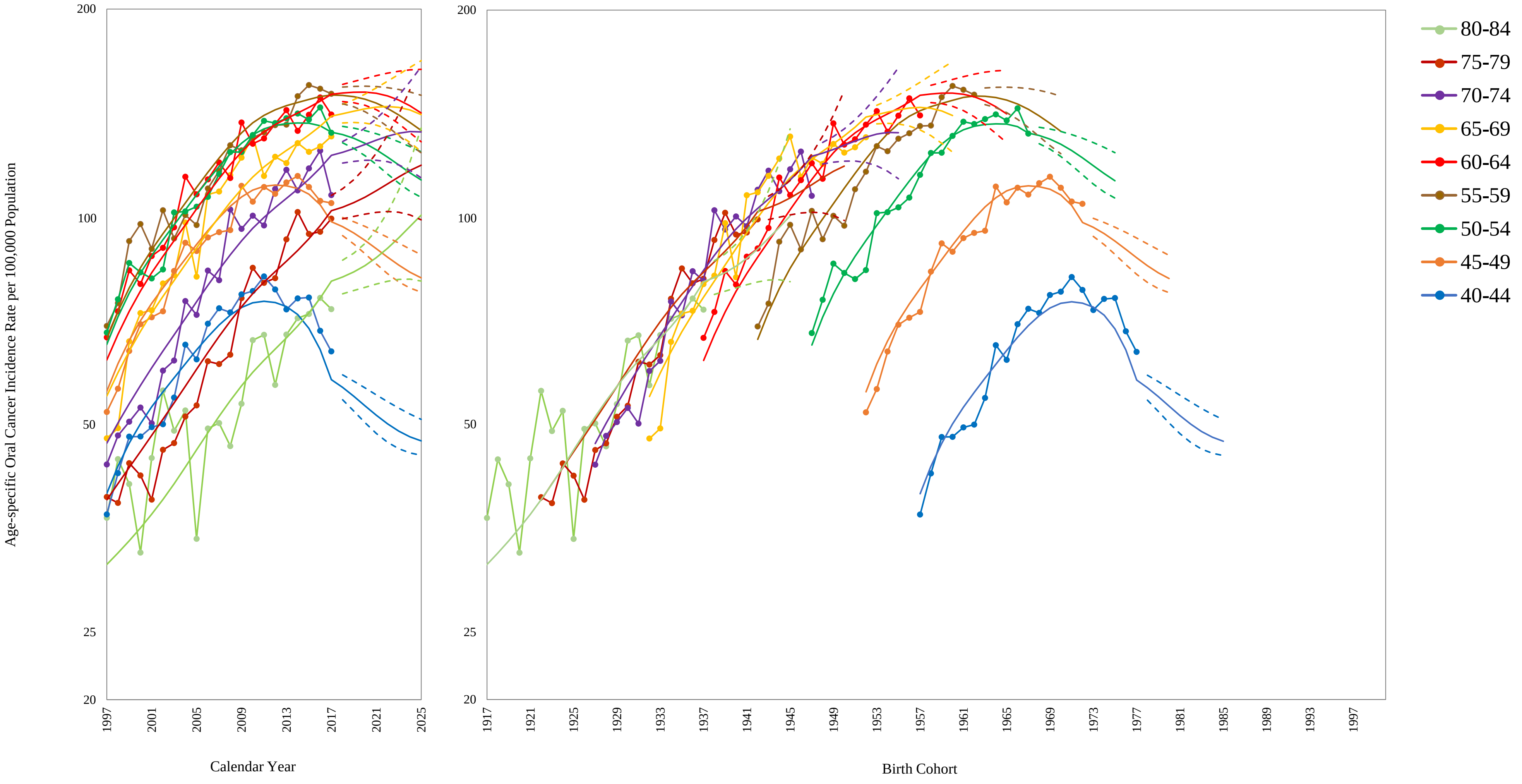

Figure S3. Age-specific oral cancer incidence rates among men in Taiwan by calendar year and birth cohort. The optimal model (from the 3-fold validation, SMAPE-T = 1.52, and LOGS = -600.10) was a polynomial model with a log link, type 3, and 75% attenuation).

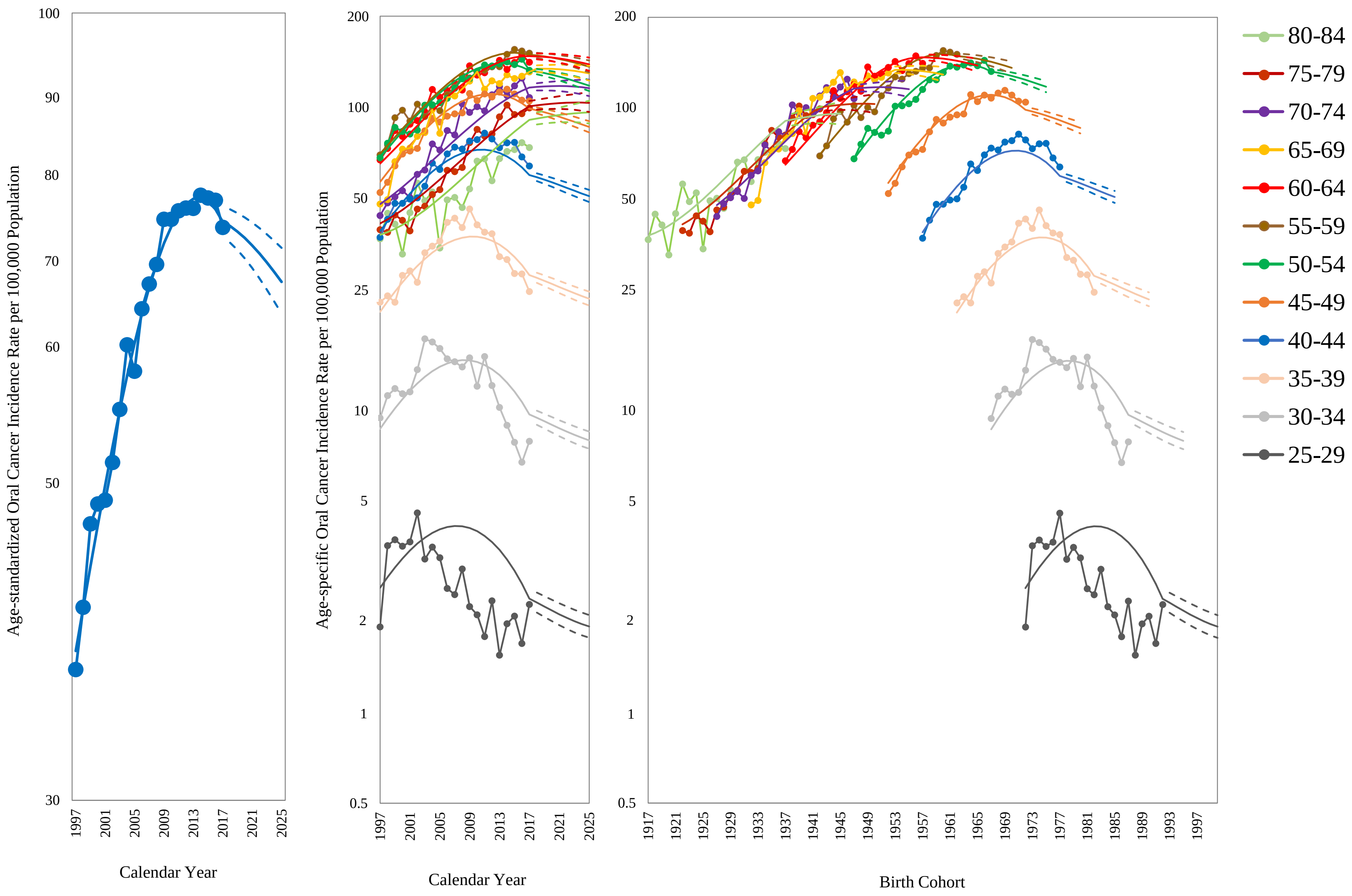

Figure S4. Age-standardized and age-specific oral cancer incidence rates among women in Taiwan by calendar year and birth cohort. The optimal model (from the 3-fold validation, SMAPE = 14.15, and LOGS = 489.46) was a polynomial model with a power 2 link, type 19, and 0% attenuation).

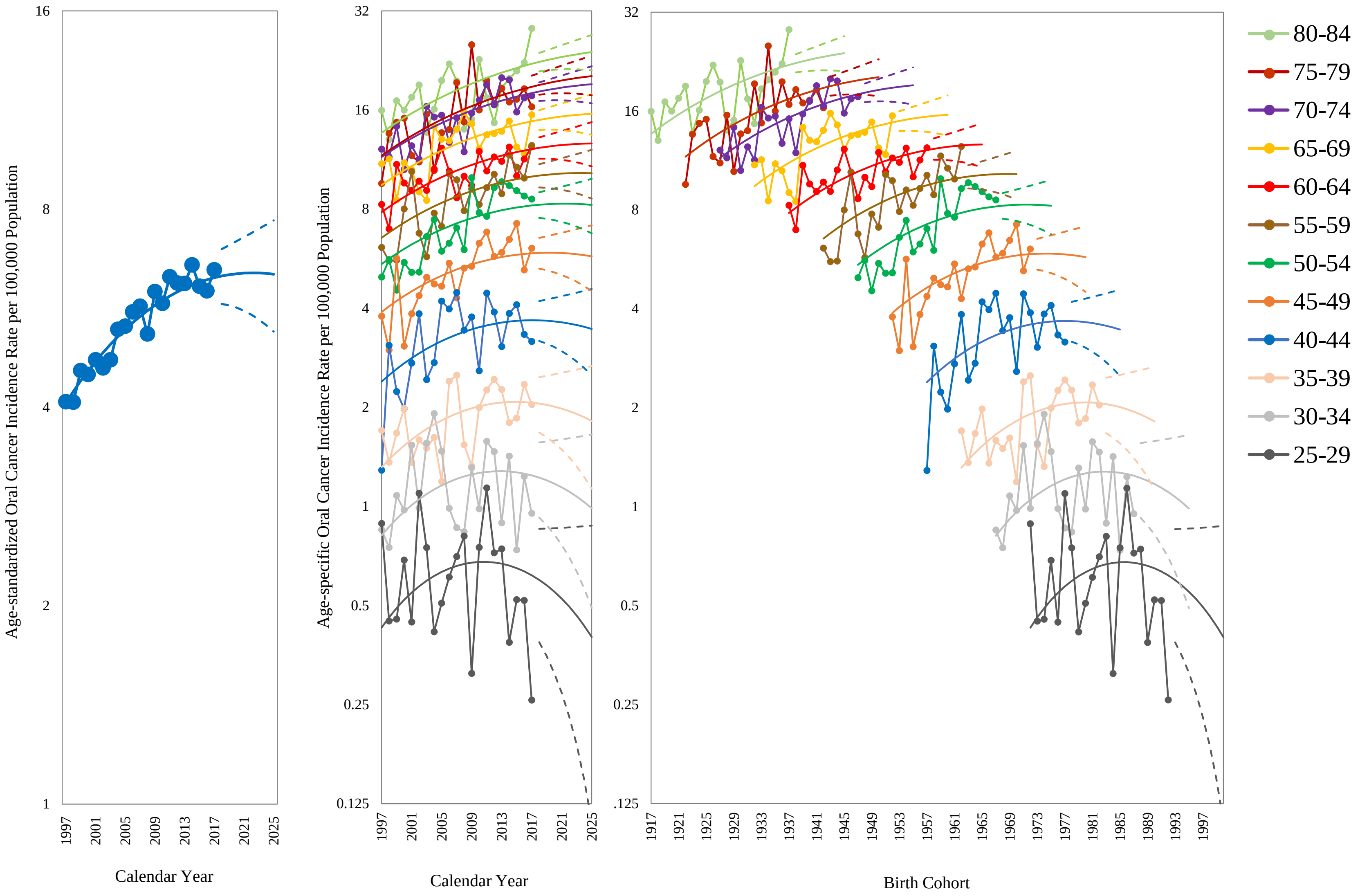

Figure S5. Age-standardized and age-specific oral cancer incidence rates among women in Taiwan by calendar year and birth cohort. The optimal model (from the 3-fold validation, SMAPE-T = 1.96, and LOGS = -273.89) was a polynomial model with a log link, type 21, and 0% attenuation).

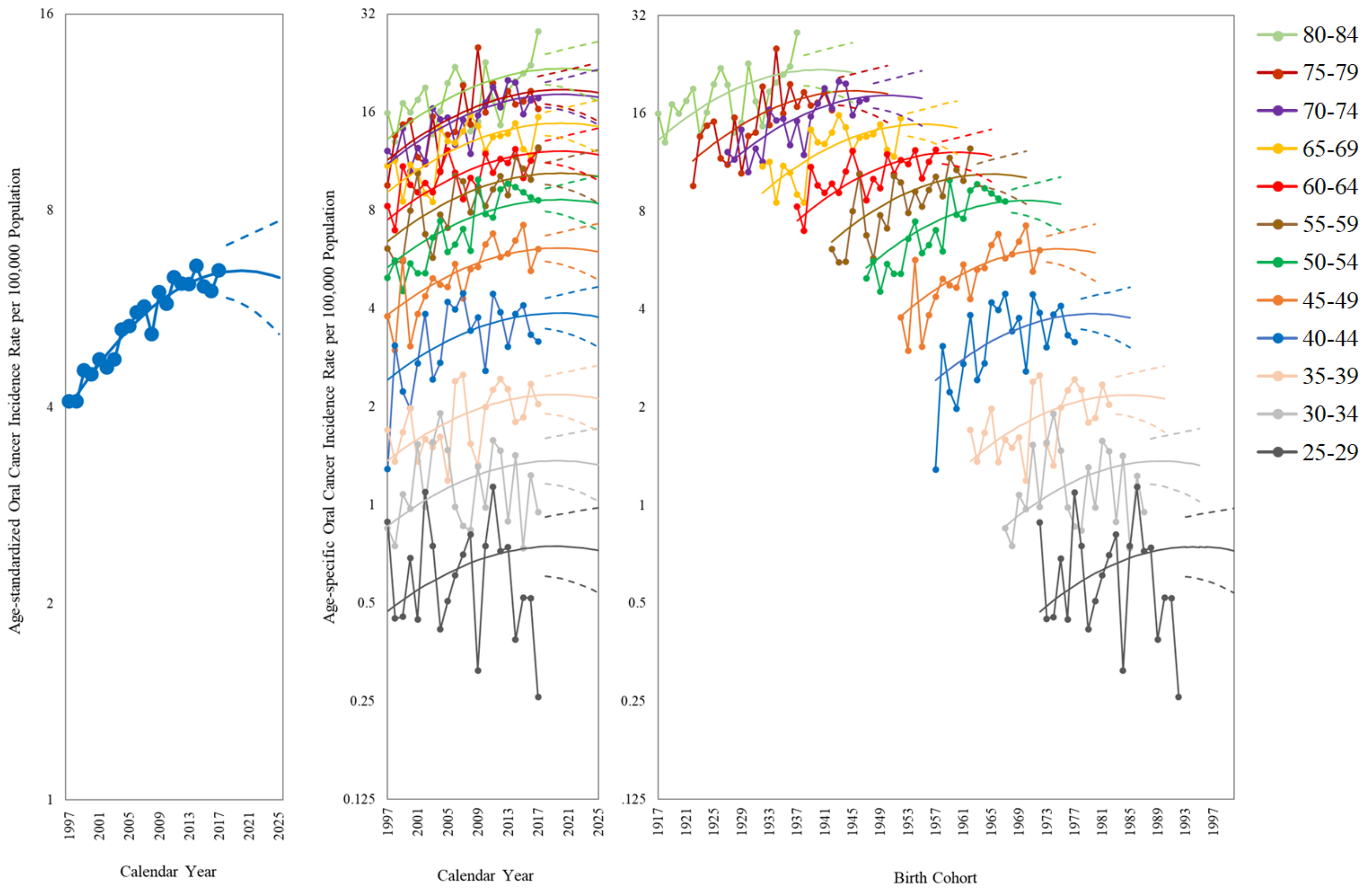

Figure S6. Age-standardized and age-specific oral cancer incidence rates among men Taiwan by calendar year and birth cohort. The optimal model (from the 5-fold validation, SMAPE = 9.27, and LOGS = 235.28) was a polynomial model with a power 5 link, type 8, and 80% attenuation).

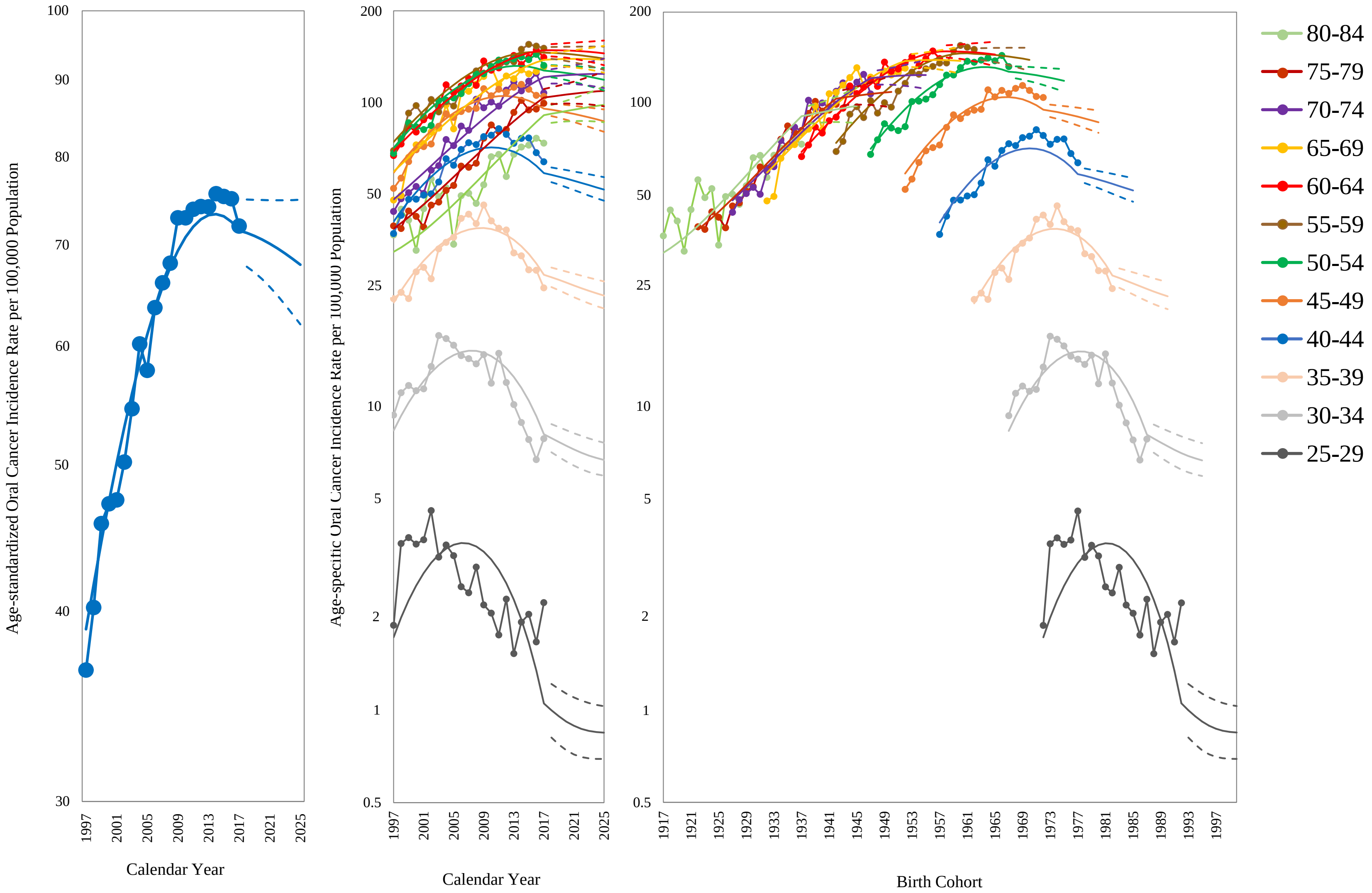

Figure S7. Age-standardized and age-specific oral cancer incidence rates among men Taiwan by calendar year and birth cohort. The optimal model (from the 5-fold validation, SMAPE-T = 1.68, and LOGS = 234.04) was a polynomial model with a power 4 link, type 3, and 90% attenuation).

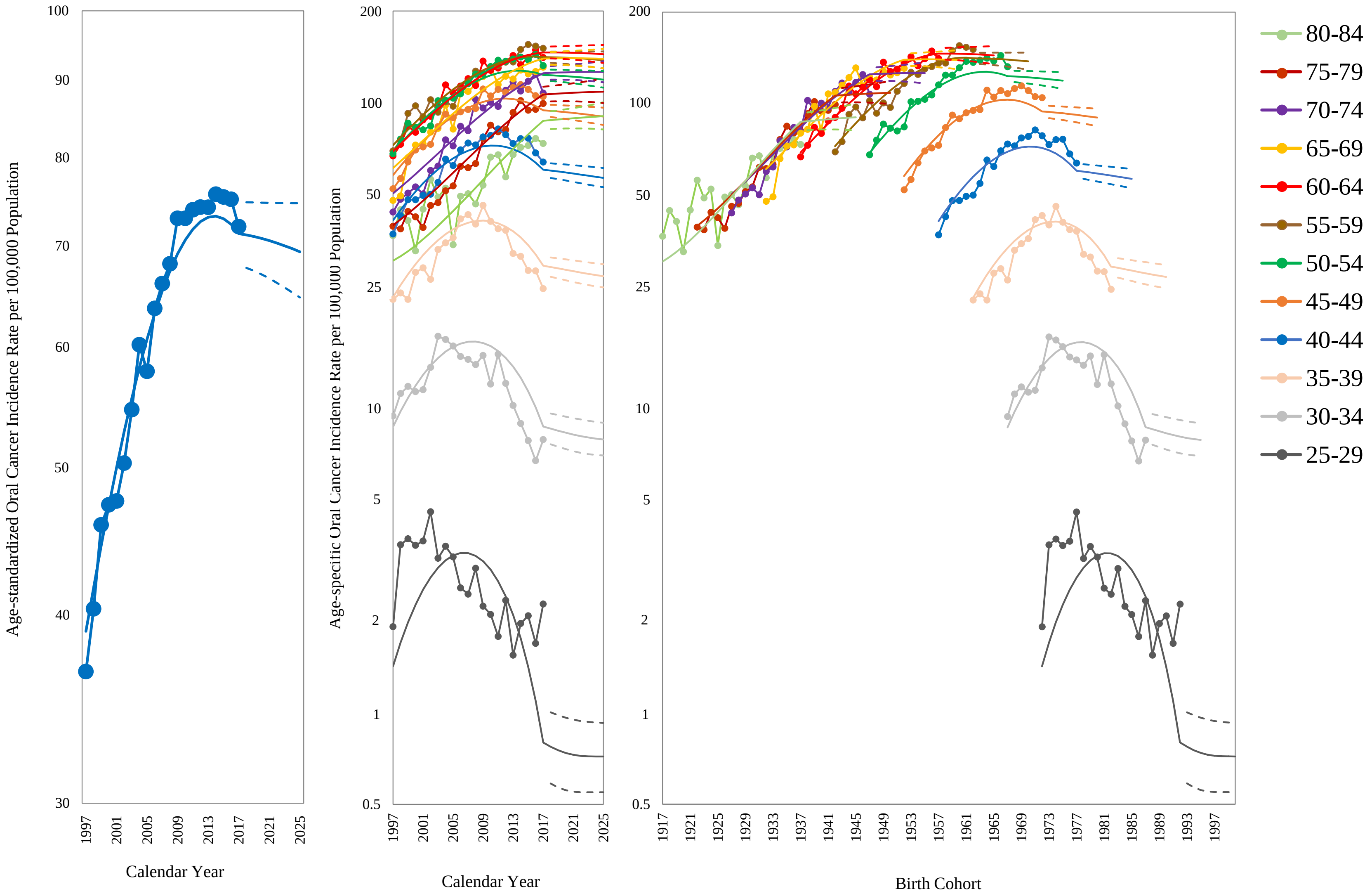

Figure S8. Age-standardized and age-specific oral cancer incidence rates among men Taiwan by calendar year and birth cohort. The optimal model (from the 3-fold validation, SMAPE = 11.52, and LOGS = -490.82) was a polynomial model with a log link, type 10, and 0% attenuation).

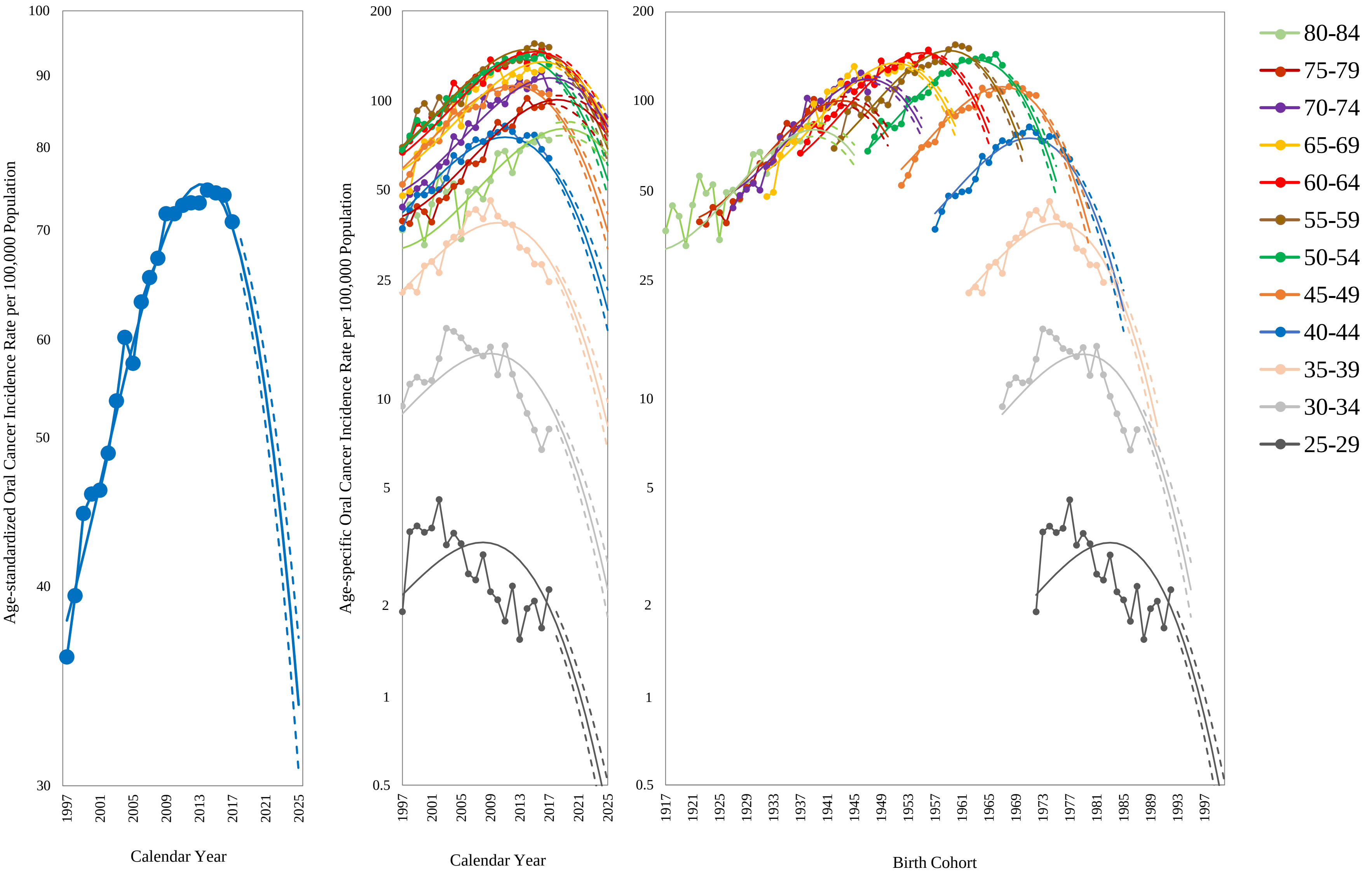

SAS tutorial for the ensemble APC approach

```
data apc;
input age period cases pop real; cards;
1 1 2 9369 2
2 1 13 9399 13
3 1 16 9800 16
4 1 36 9506 36
5 1 58 8528 58
6 1 86 6556 86
1 2 8 9554 8
2 2 12 9291 12
3 2 15 9658 15
4 2 32 9659 32
5 2 41 8684 41
6 2 95 7064 95
1 3 4 9819 4
2 3 8 9149 8
3 3 22 9557 22
4 3 34 9734 34
5 3 65 8874 65
6 3 97 7474 97
1 4 2 10076 2
2 4 4 9029 4
3 4 13 9485 13
4 4 23 9740 23
5 4 51 9028 51
6 4 104 7844 104
1 5 . 10165 3
2 5 . 9108 5
3 5 . 9393 15
4 5 . 9726 34
5 5 . 9143 57
6 5 . 8122 95
1 6 . 10183 3
2 6 . 9307 8
3 6 . 9297 17
4 6 . 9642 32
5 6 . 9292 68
6 6 . 8275 93 ;
run;

%let I=6;      /*Age*/
%let J=4;      /*Period for training set*/
%let project=2;      /*Period for validation set */

data apc_poly;
set apc2;
age2=age1**2;
age3=age1**3;
age4=age1**4;
age5=age1**5;
period2=period1**2;
period3=period1**3;
cohort1=period1-age1+&I;
cohort2=cohort1**2;
cohort3=cohort1**3;
lnpop=log(pop);
pow2rate=(cases/pop)**(1/2);
pow3rate=(cases/pop)**(1/3);
pow4rate=(cases/pop)**(1/4);
pow5rate=(cases/pop)**(1/5);
run;

proc sort data=apc_poly ;
by age1;
run;
```

SAS MACRO for the ensemble APC approach

```
%MACRO create_knot_a(k);
    %DO e=1 %TO &k;
    knot_a&e=(round(age1-(&I/%eval(&k+1))*&e))**3;
    if knot_a&e<0 then knot_a&e=0;
    %END;
%MEND;

%MACRO create_knot_p(k);
    %DO e=1 %TO &k;
    knot_p&e=(round(period1-(&J/%eval(&k+1))*&e))**3;
    if knot_p&e<0 then knot_p&e=0;
    %END;
%MEND;

%MACRO create_knot_c(k);
    %DO e=1 %TO &k;
    knot_c&e=(round(cohort1-((&I+&J-1)/%eval(&k+1))*&e))**3;
    if knot_c&e<0 then knot_c&e=0;
    %END;
%MEND;

%MACRO create_PRED(k);
    %DO e=0 %TO &k %BY 5; pred&e=(pmark-pred0)*&e/100+pred0; %END;
%MEND;

%MACRO create_SMAPE(k,trans);
    if &trans=1 then do;
        %DO e=0 %TO &k %BY 5;
        mape&e=log(100*abs(pred&e-real/pop)/((abs(pred&e)+abs(real/pop))/2)
        %END; end;
    else do;
        %DO e=0 %TO &k %BY 5;
        mape&e=(100*abs(pred&e-real/pop)/((abs(pred&e)+abs(real/pop))/2));
        %END; end;
%MEND;

%MACRO create_logP(k, dist);
    %DO e=0 %TO &k %BY 5;
    if &dist='POISSON'
    then logP&e=log(pdf('POISSON', real, pop*pred&e));
    if &dist='NORMAL'
    then logP&e=log(pdf('NORMAL',(real/pop)**pow,(pred&e)**pow, scale));
    %END;
%MEND;

%MACRO create_vars(k);
    %DO e=0 %TO &k %BY 5; mape&e logP&e %END;
%MEND;

%MACRO Select_APC(cov= , type= , res= , level= , trans= );
%LET vars=%create_vars(k=&level);

DATA input;
SET apc_poly;
    %LET K1=%SCAN(&res, 1, ' ');
    %LET K2=%SCAN(&res, 2, ' ');
    %LET K3=%SCAN(&res, 3, ' ');
    %create_knot_a(k=&K1);
    %create_knot_p(k=&K2);
    %create_knot_c(k=&K3);
RUN;

/*link=log*/
proc genmod data=input;
model cases=&cov /dist=poisson link=log offset=lnpop;
output out=poly(keep=age1 period1 real cases pop pred_cases) pred=pred_cases;
ods exclude all;
run;
```

```
proc iml;
aa=&I;
pp=&J;
cc=aa+pp-1;
L_a=(1:aa)` -    (aa/2 + 0.5);
L_p=(1:pp)` -    (pp/2 + 0.5);
L_c=(1:cc)` -    (cc/2 + 0.5);
   temp1=j(aa,1,1);
   temp2=L_a;
   do i=2 to aa;
        temp1=temp1||temp2;
        temp2=temp2#L_a;
        end;
   call gsorth(X_a,t,lin,temp1);
   temp1=j(pp,1,1);
   temp2=L_p;
   do i=2 to pp;
        temp1=temp1||temp2;
        temp2=temp2#L_p;
        end;
   call gsorth(X_p,t,lin,temp1);
   temp1=j(cc,1,1);
   temp2=L_c;
   do i=2 to cc;
        temp1=temp1||temp2;
        temp2=temp2#L_c;
        end;
   call gsorth(X_c,t,lin,temp1);
   X_a=(1:aa)`||X_a[,3:aa];
   X_p=(1:pp)`||X_p[,3:pp];
   X_c=(1:cc)`||X_c[,3:cc];
   aname="a0":"a%eval(&I-2)";
   pname="p0":"p%eval(&J-2)";
   cname="c0":"c%eval(&I+&J-3)";
create X_a from X_a [colname=aname];
append from X_a;
create X_p from X_p [colname=pname];
append from X_p;
create X_c from X_c [colname=cname];
append from X_c;
run;
quit;

proc sort data=X_a out=X_a (rename=a0=age1);
by a0;
run;

/*orthogonal age*/
data apc_poly;
merge apc_poly X_a;
by age1;
run;

proc sort data=apc_poly;
by period1 age1;
run;


%Select_APC(cov=age1-age2 period1 period2 cohort2,
type=Type1);
%Select_APC(cov=age1-age2 period1 cohort2,
type=Type2);
%Select_APC(cov=age1-age3 period1-period3 cohort2
cohort3, type=Type3);
%Select_APC(cov=age1-age3 period1-period3 cohort2,
type=Type4);
%Select_APC(cov=age1-age3 period1 period2 cohort2
cohort3, type=Type5);
%Select_APC(cov=age1-age3 period1 period2 cohort2,
type=Type6);
```

```
data ppp(keep=age1 pmark); set poly;
pmark=pred_cases/pop;
if period1=&J;
run;

proc sort data=ppp; by age1; run;
proc sort data=poly; by age1; run;
data attenuation; merge poly ppp; by age1; run;

proc sort data=attenuation; by period1 age1; run;
data attenuation; set attenuation;
pred0=pred_cases/pop;
if period1<=&J then do;pmark=.;pred0=.;end;
%create_PRED(k=&level);
run;

data mape; set attenuation;
if period1<=&J then do;pmark=.;pred0=.;end;
%create_SMAPE(k=&level,trans=&trans);
%create_logP(k=&level,dist='POISSON');
run;

proc means data=mape NOPRINT;
var &vars;
output out=temp;
run;

DATA TEMP; LENGTH ID $8; SET TEMP; ID='log'; RUN;

/*link=power 2*/
proc genmod data=input;
model pow2rate=&cov /dist=normal link=identity;
output out=poly(keep=age1 period1 real cases pop pred_cases) pred=pred_cases;
ods output ParameterEstimates=parms;
run;

DATA _NULL_; SET parms;
IF Parameter='scale' THEN DO; call symput('scale',Estimate); END;
run; ods exclude all;

data ppp(keep=age1 pmark); set poly;
pmark=((pred_cases)**2);
if period1=&J;
run;

proc sort data=ppp; by age1; run;
proc sort data=poly; by age1; run;
data attenuation;
merge poly ppp; by age1;
pred=((pred_cases)**2);
run;

proc sort data=attenuation; by period1 age1; run;
data attenuation; set attenuation;
pred0=((pred_cases)**2);
if period1<=&J then do;pmark=.;pred0=.;end;
%create_PRED(k=&level);
run;

data mape; set attenuation;
if period1<=&J then do;pmark=.;pred0=.;end;
scale=&scale; pow=1/2;
%create_SMAPE(k=&level,trans=&trans);
%create_logP(k=&level,dist='NORMAL');
run;

proc means data=mape NOPRINT;
var &vars; output out=temp1; run;

DATA TEMP; SET TEMP temp1(in=A); IF A THEN ID='power2'; RUN;
```

```
%Select_APC(cov=age1-age3 period1 cohort2, type=Type7);
%Select_APC(cov=age1-age4 period1-period3 cohort2
cohort3, type=Type8);
%Select_APC(cov=age1-age4 period1 period2 period3
cohort2, type=Type9);
%Select_APC(cov=age1-age4 period1 period2 cohort2
cohort3, type=Type10);
%Select_APC(cov=age1-age4 period1 period2 cohort2,
type=Type11);
%Select_APC(cov=age1-age4 period1 cohort2,
type=Type12);
%Select_APC(cov=age1-age5 period1-period3 cohort2
cohort3, type=Type13);
%Select_APC(cov=age1-age5 period1-period3 cohort2,
type=Type14);
%Select_APC(cov=age1-age5 period1 period2 cohort2
cohort3, type=Type15);
%Select_APC(cov=age1-age5 period1 period2 cohort2,
type=Type16);
%Select_APC(cov=age1-age5 period1 cohort2,
type=Type17);
%Select_APC(cov=age1 a1-a10 period1-period3 cohort2
cohort3, type=Type18);
%Select_APC(cov=age1 a1-a10 period1-period3 cohort2,
type=Type19);
%Select_APC(cov=age1 a1-a10 period1 period2 cohort2
cohort3, type=Type20);
%Select_APC(cov=age1 a1-a10 period1 period2 cohort2,
type=Type21);
%Select_APC(cov=age1 a1-a10 period1 cohort2,
type=Type22);
%Select_APC(cov=age1-age3 knot_a1 knot_a2
period1-period3 knot_p1 knot_p2 cohort2 cohort3 knot_c1
knot_c2, type=Type23, res=2 2 2);
%Select_APC(cov=age1-age3 knot_a1-knot_a3
period1-period3 knot_p1 knot_p2 cohort2 cohort3 knot_c1
knot_c2, type=Type24, res=3 2 2);
%Select_APC(cov=age1-age3 knot_a1-knot_a4
period1-period3 knot_p1 knot_p2 cohort2 cohort3 knot_c1
knot_c2, type=Type25, res=4 2 2);
%Select_APC(cov=age1-age3 knot_a1 knot_a2
period1-period3 knot_p1 knot_p2 cohort2 cohort3
knot_c1-knot_c3, type=Type26, res=2 2 3);
%Select_APC(cov=age1-age3 knot_a1-knot_a3
period1-period3 knot_p1 knot_p2 cohort2 cohort3
knot_c1-knot_c3, type=Type27, res=3 2 3);
%Select_APC(cov=age1-age3 knot_a1-knot_a4
period1-period3 knot_p1 knot_p2 cohort2 cohort3
knot_c1-knot_c3, type=Type28, res=4 2 3);
%Select_APC(cov=age1-age3 knot_a1 knot_a2
period1-period3 knot_p1 knot_p2 cohort2 cohort3
knot_c1-knot_c4, type=Type29, res=2 2 4);
%Select_APC(cov=age1-age3 knot_a1-knot_a3
period1-period3 knot_p1 knot_p2 cohort2 cohort3
knot_c1-knot_c4, type=Type30, res=3 2 4);
%Select_APC(cov=age1-age3 knot_a1-knot_a4
period1-period3 knot_p1 knot_p2 cohort2 cohort3
knot_c1-knot_c4, type=Type31, res=4 2 4);
%Select_APC(cov=age1-age3 knot_a1 knot_a2
period1-period3 knot_p1 knot_p2 cohort2 cohort3
knot_c1-knot_c5, type=Type32, res=2 2 5);
%Select_APC(cov=age1-age3 knot_a1-knot_a3
period1-period3 knot_p1 knot_p2 cohort2 cohort3
knot_c1-knot_c5, type=Type33, res=3 2 5);
%Select_APC(cov=age1-age3 knot_a1-knot_a4
period1-period3 knot_p1 knot_p2 cohort2 cohort3
knot_c1-knot_c5, type=Type34, res=4 2 5);
%Select_APC(cov=age1-age3 knot_a1 knot_a2
period1-period3 knot_p1 knot_p2 cohort2 cohort3
knot_c1-knot_c6, type=Type35, res=2 2 6);
```

```
/*link=power 3*/
proc genmod data=input;
model pow3rate=&cov/ dist=normal link=identity;
output out=poly(keep=age1 period1 real cases pop pred_cases) pred=pred_cases;
ods output ParameterEstimates=parms;
run;

DATA _NULL_; SET parms;
IF Parameter='scale' THEN DO; call symput('scale',Estimate); END;
run; ods exclude all;

data ppp(keep=age1 pmark); set poly;
pmark=((pred_cases)**3);
if period1=&J;
run;

proc sort data=ppp; by age1; run;
proc sort data=poly; by age1; run;
data attenuation;
merge poly ppp; by age1;
pred=((pred_cases)**3);
run;

proc sort data=attenuation; by period1 age1; run;
data attenuation; set attenuation;
pred0=((pred_cases)**3);
if period1<=&J then do;pmark=.;pred0=.;end;
%create_PRED(k=&level);
run;

data mape; set attenuation;
if period1<=&J then do;pmark=.;pred0=.;end;
scale=&scale; pow=1/3;
%create_SMAPE(k=&level,trans=&trans);
%create_logP(k=&level,dist='NORMAL');
run;

proc means data=mape NOPRINT;
var &vars;
output out=temp1;
run;

DATA TEMP; SET TEMP temp1(in=A); IF A THEN ID='power3'; RUN;

/*link=power 4*/
proc genmod data=input;
model pow4rate=&cov /dist=normal link=identity;
output out=poly(keep=age1 period1 real cases pop pred_cases) pred=pred_cases;
ods output ParameterEstimates=parms;
run;

DATA _NULL_; SET parms;
IF Parameter='scale' THEN DO; call symput('scale',Estimate); END;
run; ods exclude all;

data ppp(keep=age1 pmark); set poly;
pmark=((pred_cases)**4);
if period1=&J;
run;

proc sort data=ppp; by age1; run;
proc sort data=poly; by age1; run;
data attenuation;
merge poly ppp; by age1;
pred=((pred_cases)**4);
run;

proc sort data=attenuation; by period1 age1; run;
data attenuation; set attenuation;
pred0=((pred_cases)**4);
```

```
%Select_APC(cov=age1-age3 knot_a1-knot_a3
period1-period3 knot_p1 knot_p2 cohort2 cohort3
knot_c1-knot_c6, type=Type36, res=3 2 6);
%Select_APC(cov=age1-age3 knot_a1-knot_a4
period1-period3 knot_p1 knot_p2 cohort2 cohort3
knot_c1-knot_c6, type=Type37, res=4 2 6);
%Select_APC(cov=age1-age3 knot_a1 knot_a2
period1-period3 knot_p1-knot_p3 cohort2 cohort3 knot_c1
knot_c2, type=Type38, res=2 3 2);
%Select_APC(cov=age1-age3 knot_a1-knot_a3
period1-period3 knot_p1-knot_p3 cohort2 cohort3 knot_c1
knot_c2, type=Type39, res=3 3 2);
%Select_APC(cov=age1-age3 knot_a1-knot_a4
period1-period3 knot_p1-knot_p3 cohort2 cohort3 knot_c1
knot_c2, type=Type40, res=4 3 2);
%Select_APC(cov=age1-age3 knot_a1 knot_a2
period1-period3 knot_p1-knot_p3 cohort2 cohort3
knot_c1-knot_c3, type=Type41, res=2 3 3);
%Select_APC(cov=age1-age3 knot_a1-knot_a3
period1-period3 knot_p1-knot_p3 cohort2 cohort3
knot_c1-knot_c3, type=Type42, res=3 3 3);
%Select_APC(cov=age1-age3 knot_a1-knot_a4
period1-period3 knot_p1-knot_p3 cohort2 cohort3
knot_c1-knot_c3, type=Type43, res=4 3 3);
%Select_APC(cov=age1-age3 knot_a1 knot_a2
period1-period3 knot_p1-knot_p3 cohort2 cohort3
knot_c1-knot_c4, type=Type44, res=2 3 4);
%Select_APC(cov=age1-age3 knot_a1-knot_a3
period1-period3 knot_p1-knot_p3 cohort2 cohort3
knot_c1-knot_c4, type=Type45, res=3 3 4);
%Select_APC(cov=age1-age3 knot_a1-knot_a4
period1-period3 knot_p1-knot_p3 cohort2 cohort3
knot_c1-knot_c4, type=Type46, res=4 3 4);
%Select_APC(cov=age1-age3 knot_a1 knot_a2
period1-period3 knot_p1-knot_p3 cohort2 cohort3
knot_c1-knot_c5, type=Type47, res=2 3 5);
%Select_APC(cov=age1-age3 knot_a1-knot_a3
period1-period3 knot_p1-knot_p3 cohort2 cohort3
knot_c1-knot_c5, type=Type48, res=3 3 5);
%Select_APC(cov=age1-age3 knot_a1-knot_a4
period1-period3 knot_p1-knot_p3 cohort2 cohort3
knot_c1-knot_c5, type=Type49, res=4 3 5);
%Select_APC(cov=age1-age3 knot_a1 knot_a2
period1-period3 knot_p1-knot_p3 cohort2 cohort3
knot_c1-knot_c6, type=Type50, res=2 3 6);
%Select_APC(cov=age1-age3 knot_a1-knot_a3
period1-period3 knot_p1-knot_p3 cohort2 cohort3
knot_c1-knot_c6, type=Type51, res=3 3 6);
%Select_APC(cov=age1-age3 knot_a1-knot_a4
period1-period3 knot_p1-knot_p3 cohort2 cohort3
knot_c1-knot_c6, type=Type52, res=4 3 6);

DATA result;
SET Type1-Type52;
MAPE=min(mape0, mape5, mape10, mape15, mape20,
mape25, mape30, mape35, mape40, mape45, mape50,
mape55, mape60, mape65, mape70, mape75, mape80,
mape85, mape90, mape95, mape100);
logP=max(logP0, logP5, logP10, logP15, logP20, logP25,
logP30, logP35, logP40, logP45, logP50, logP55, logP60,
logP65, logP70,logP75, logP80, logP85, logP90, logP95,
logP100);

PROC SORT; BY MAPE; RUN;
```

```
if period1<=&J then do;pmark=.;pred0=.;end;
%create_PRED(k=&level);
run;

data mape; set attenuation;
if period1<=&J then do;pmark=.;pred0=.;end;
scale=&scale; pow=1/4;
%create_SMAPE(k=&level,trans=&trans);
%create_logP(k=&level,dist='NORMAL');
run;

proc means data=mape NOPRINT;
var &vars;
output out=temp1;
run;

DATA TEMP; SET TEMP temp1(in=A); IF A THEN ID='power4'; RUN;

/*link=power 5*/
proc genmod data=input;
model pow5rate=&cov /dist=normal link=identity;
output out=poly(keep=age1 period1 real cases pop pred_cases) pred=pred_cases;
ods output ParameterEstimates=parms;
run;

DATA _NULL_; SET parms;
IF Parameter='scale' THEN DO; call symput('scale',Estimate); END;
run; ods exclude all;

data ppp(keep=age1 pmark); set poly;
pmark=((pred_cases)**5);
if period1=&J;
run;

proc sort data=ppp; by age1; run;
proc sort data=poly; by age1; run;
data attenuation;
merge poly ppp; by age1;
pred=((pred_cases)**5);
run;

proc sort data=attenuation; by period1 age1; run;
data attenuation; set attenuation;
pred0=((pred_cases)**5);
if period1<=&J then do;pmark=.;pred0=.;end;
%create_PRED(k=&level);
run;

data mape;
set attenuation;
if period1<=&J then do;pmark=.;pred0=.;end;
scale=&scale; pow=1/5;
%create_SMAPE(k=&level,trans=&trans);
%create_logP(k=&level,dist='NORMAL');
run;

proc means data=mape NOPRINT;
var &vars;
output out=temp1;
run;

DATA &type; LENGTH TYPE $10.;
SET TEMP temp1(in=A);
TYPE="&type";
IF A THEN ID='power5';
IF _STAT_='MEAN';
DROP _FREQ_ _STAT_ _TYPE_ ;
RUN;
%MEND;
```
